# The Sphingolipid Receptor S1PR2 Is a Receptor for Nogo-A Repressing Synaptic Plasticity

**DOI:** 10.1371/journal.pbio.1001763

**Published:** 2014-01-14

**Authors:** Anissa Kempf, Bjoern Tews, Michael E. Arzt, Oliver Weinmann, Franz J. Obermair, Vincent Pernet, Marta Zagrebelsky, Andrea Delekate, Cristina Iobbi, Ajmal Zemmar, Zorica Ristic, Miriam Gullo, Peter Spies, Dana Dodd, Daniel Gygax, Martin Korte, Martin E. Schwab

**Affiliations:** 1 Brain Research Institute, University of Zurich, and Dept. of Health Sciences and Technology, Swiss Federal Institute of Technology, Zurich, Switzerland; 2 Zoological Institute, Division of Cellular Neurobiology, TU Braunschweig, Braunschweig, Germany; 3 School of Life Sciences, University of Applied Life Sciences Northwestern Switzerland, Muttenz, Switzerland; London Research Institute, United Kingdom

## Abstract

Nogo-A is a membrane protein of the central nervous system (CNS) restricting neurite growth and synaptic plasticity via two extracellular domains: Nogo-66 and Nogo-A-Δ20. Receptors transducing Nogo-A-Δ20 signaling remained elusive so far. Here we identify the G protein-coupled receptor (GPCR) sphingosine 1-phosphate receptor 2 (S1PR2) as a Nogo-A-Δ20-specific receptor. Nogo-A-Δ20 binds S1PR2 on sites distinct from the pocket of the sphingolipid sphingosine 1-phosphate (S1P) and signals via the G protein G_13_, the Rho GEF LARG, and RhoA. Deleting or blocking S1PR2 counteracts Nogo-A-Δ20- and myelin-mediated inhibition of neurite outgrowth and cell spreading. Blockade of S1PR2 strongly enhances long-term potentiation (LTP) in the hippocampus of wild-type but not Nogo-A^−/−^ mice, indicating a repressor function of the Nogo-A/S1PR2 axis in synaptic plasticity. A similar increase in LTP was also observed in the motor cortex after S1PR2 blockade. We propose a novel signaling model in which a GPCR functions as a receptor for two structurally unrelated ligands, a membrane protein and a sphingolipid. Elucidating Nogo-A/S1PR2 signaling platforms will provide new insights into regulation of synaptic plasticity.

## Abstract 2

This study identifies a GPCR, S1PR2, as a receptor for the Nogo-A-Δ20 domain of the membrane protein Nogo-A, which inhibits neuronal growth and synaptic plasticity.

## Author Summary

Recent studies have demonstrated an important role of Nogo-A signaling in the repression of structural and synaptic plasticity in mature neuronal networks of the central nervous system. These insights extended our understanding of Nogo-A's inhibitory function far beyond its well-studied role as axonal-growth inhibitor. Repression is mediated via two different Nogo-A extracellular domains: Nogo-66 and Nogo-A-Δ20. Here, we identify the G-protein coupled receptor S1PR2 as a high-affinity receptor for Nogo-A-Δ20 and demonstrate that S1PR2 binds this domain with sites different from the recently proposed S1P binding pocket. Interfering with S1PR2 activity, either pharmacologically or genetically, prevented Nogo-A-Δ20-mediated inhibitory effects. Similar results were obtained when we blocked G_13_, LARG, and RhoA, components of the downstream signaling pathway. These findings revealed a strong increase in hippocampal and cortical synaptic plasticity when acutely interfering with Nogo-A/S1PR2 signaling, similar to previous results obtained by blocking Nogo-A. We thus provide a novel biological concept of multi-ligand GPCR signaling in which this sphingolipid-activated GPCR is also bound and activated by the high molecular weight membrane protein Nogo-A.

## Introduction

Factors inhibiting nerve fiber growth substantially contribute to the limited regenerative capacity of the adult central nervous system (CNS) after injury. They play important roles in stabilizing the complex wiring of the adult CNS of higher vertebrates and in establishing neuronal pathways in the developing nervous system [1],[2]. One of the best-studied factors is the membrane protein Nogo-A, which occurs in myelin and certain neurons, inhibiting axonal regeneration and plasticity after CNS injury [3]–[5]. Neutralization of Nogo-A has been shown to enhance axonal growth and compensatory sprouting in the adult spinal cord and brain, as well as to improve functional recovery after CNS injury [4],[6]. Recent studies have shown novel important roles of Nogo-A signaling in the repression of synaptic plasticity in mature neuronal networks, indicating an inhibitory potential of Nogo-A far beyond its well-studied restriction of axonal growth [1],[7]–[11].

Nogo-A exerts its inhibitory effects via two distinct extracellular domains: Nogo-66 (rat amino acid (aa) 1026–1091) and Nogo-A-Δ20 (rat aa544–725; part of “Amino-Nogo”) [2],[12]. Nogo-66 induces growth inhibition via two membrane proteins, Nogo-66 receptor 1 (NgR1) [13], together with accessory proteins, and paired immunoglobulin-like receptor B (PirB) [14]. By contrast, the molecular identification and characterization of the receptor(s) transducing signals from the inhibitory Nogo-A-Δ20 domain has failed so far [2]. Nogo-A-Δ20 has been shown to partially mediate its inhibitory activity by interfering with integrins, but proof of a direct interaction has remained elusive [15]. Here we identified the G protein-coupled receptor (GPCR) sphingosine 1-phosphate receptor 2 (S1PR2) as a functional receptor for the Δ20 domain of Nogo-A.

S1PR2 belongs to the subfamily of five S1PRs [16]. S1PRs are known to be activated by the low molecular weight (MW) lipid ligand sphingosine 1-phosphate (S1P), which exerts diverse receptor-specific effects on various cell types, including regulation of apoptosis, cell motility and cytoskeleton dynamics [16]. In the brain and spinal cord, S1P has been shown to regulate angiogenesis and neurite outgrowth: activation of S1PR1 promotes neurite outgrowth *in vitro* via G_i/o_ and Rac1, whereas activation of S1PR2 leads to neurite retraction, involving G_i/o_, G_q_, or G_12/13_ and the RhoA pathway [16]–[18].

In this study we demonstrate that Nogo-A-Δ20 binds S1PR2 *via* extracellular receptor loops 2 and 3, which are distinct from the previously described binding site of S1P [19]. Nogo-A-Δ20 signals through the G protein G_13_, leukemia-associated Rho guanine exchange factor (RhoGEF) LARG and RhoA. Deleting or blocking S1PR2 counteracts Nogo-A-Δ20- and myelin-mediated inhibition of neurite outgrowth and cell spreading. Acute S1PR2 blockade increases hippocampal and cortical long-term synaptic plasticity similarly to Nogo-A neutralization. These results strengthen the recently proposed physiological role of Nogo-A in restricting synaptic plasticity to stabilize neuronal circuits [1],[9]. Further, these data support the paradigm shift for GPCR signaling from the classical “one ligand – one receptor” situation towards more dynamic models [20],[21].

## Results

### Nogo-A Binds to S1PR2

**Figure 1:**
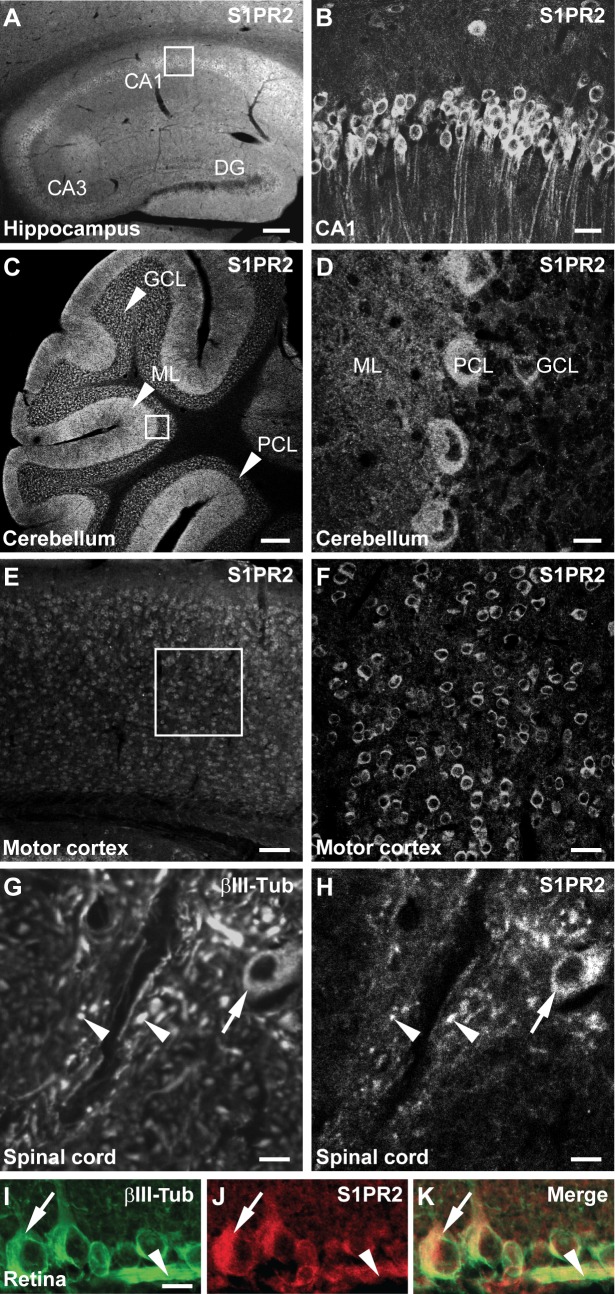
Localization of S1PR2 by immunohistochemistry in the adult mouse CNS. (A) S1PR2 expression in the hippocampus. CA, *cornu ammonis*; DG, dentate gyrus. (B) Magnification of the boxed region of CA1 depicted in (A). (C) S1PR2 expression in the cerebellum. GCL, granule cell layer; ML, molecular layer; PCL, Purkinje cell layer. (D) Magnification of the boxed region depicted in (C). (E) S1PR2 expression in the motor cortex. (F) Magnification of the boxed region depicted in (E). (G,H) S1PR2 expression in motoneuron cell bodies (arrows) and βIII-Tubulin-positive fibers (arrowheads) in the spinal cord. (I,J,K) S1PR2 expression in βIII-Tubulin-positive axons bundles (arrowheads) and cell bodies (arrows) of retinal ganglion cells. Scale bars: (A) 300 µm; (B) 30 µm; (C) 200 µm; (D) 15 µm; (E) 90 µm; (F) 30 µm; (G,H) 20 µm; (I–K) 15 µm.

**Figure 2:**
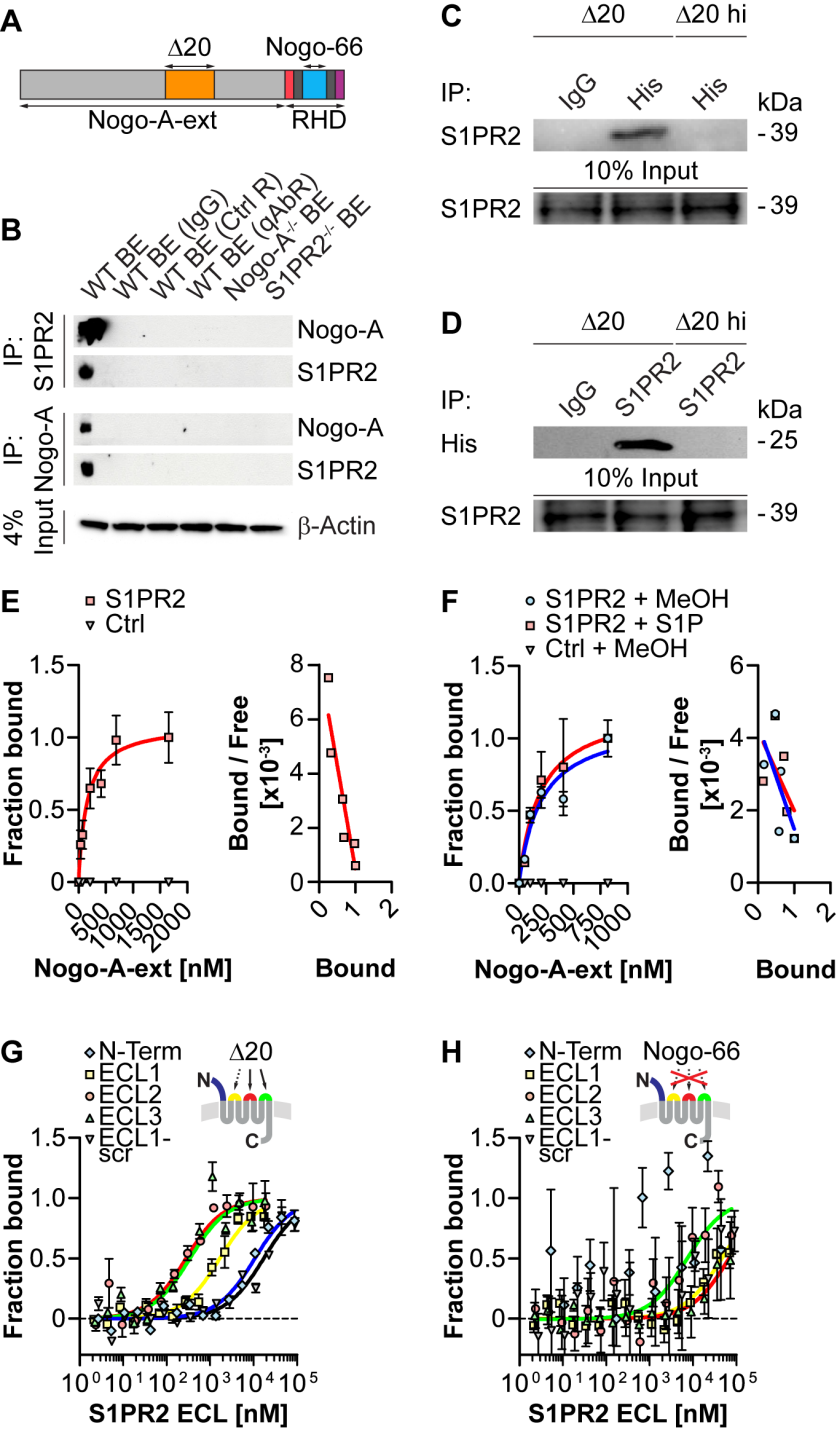
Nogo-A binds to S1PR2. (A) Schematic structure of Nogo-A showing the inhibitory domains Nogo-A-Δ20 (Δ20, orange), Nogo-66 (blue), and Nogo-A-ext. Transmembrane domains are indicated in dark grey. RHD, reticulon homology domain. (B) Nogo-A (∼200 kDa) co-immunoprecipitated with S1PR2 (∼40 kDa) and vice-versa in WT but not Nogo-A^−/−^ or S1PR2^−/−^ brain extracts (BE). If specified, the following controls were used in WT BE instead of the IP antibody to confirm the specificity of the interaction: IgG, control antibody; Ctrl R, resin only control; qAbR, quenched antibody (Ab) resin control. Input loading control: β-Actin (∼42 kDa). (C) S1PR2 immunoprecipitated with His-tagged Δ20 but not heat-inactivated (hi) Δ20 in S1PR2-overexpressing membranes. Input loading control: S1PR2. (D) His-tagged Δ20 but not hi Δ20 immunoprecipitated with S1PR2 in S1PR2-overexpressing membranes. Input loading control: S1PR2. (E) Nogo-A-ext bound specifically to biosensor-immobilized S1PR2-overexpressing versus control membranes (*K*_D_∼142 nM). A Scatchard plot analysis is shown on the right. (F) 1 µM S1P does not modulate the interaction between Nogo-A-ext and S1PR2 when compared to the methanol (MeOH) vehicle control (MeOH, *K*_D_∼192 nM; S1P, *K*_D_∼202 nM). A Scatchard plot analysis is shown on the right. (G) Microscale thermophoresis binding analysis of Δ20 to S1PR2 extracellular domains: ECL2 (*K*_D_∼280 nM), ECL3 (*K*_D_∼350 nM), ECL1 (*K*_D_∼1.7 µM), and N-terminus (*K*_D_∼11 µM). Scrambled ECL1 (ECL1-scr) was used as control (*K*_D_∼17 µM). Arrows indicate the identified Δ20-binding loops in S1PR2. (H) Nogo-66 binding to S1PR2 extracellular domains is unspecific: ECL2 (*K*_D_∼7 µM), ECL1 (*K*_D_∼46 µM), ECL3 (*K*_D_∼67 µM). No binding to the N-Terminus or to ECL1-scr is observed.

The GPCR S1PR2 was identified as a novel receptor candidate of the Nogo-A-Δ20 domain using a yeast two-hybrid (Y2H) screen of custom-made adult and fetal human brain libraries. In the adult CNS, S1PR2 is mainly expressed in the grey matter (Figure 1). Hippocampal pyramidal cells, cerebellar Purkinje cells, cortical neurons and spinal motoneurons, as well as retinal ganglion cells are S1PR2-positive (Figure 1A–1K). Importantly, S1PR2 is also expressed in Nogo-A-Δ20-responsive cells *in vitro* including 3T3 fibroblasts and immature cerebellar granule neurons (Figure S1). To validate the interaction of Nogo-A-Δ20 (Figure 2A) and S1PR2, His-tagged Nogo-A-Δ20 was co-incubated with membranes of S1PR2-overexpressing cells and subsequently immunoprecipitated (Figure 2C). S1PR2 was specifically detected in immunoprecipitation fractions (Figure 2C). Vice versa, His-tagged Nogo-A-Δ20 could be specifically probed in S1PR2 immunoprecipitated fractions, suggesting that the two proteins interact *in vitro* (Figure 2D). Co-immunoprecipitation experiments of Nogo-A or S1PR2 from whole mouse brain protein extracts further demonstrated that endogenous S1PR2 interacts with Nogo-A under physiological conditions *in vivo* (Figure 2B). To determine the binding affinity, binding of the entire Δ20-containing extracellular N-terminal domain of Nogo-A (Nogo-A-ext; Figure 2A) to biosensor-immobilized membrane preparations expressing functional full length S1PR2 protein or non S1PR2-expressing control membranes was monitored in real-time using Bio-Layer interferometry (OctetRED). Non-linear fitting revealed that Nogo-A-ext binds to S1PR2 with an apparent equilibrium binding constant (*K*_D_) of ∼142 nM (Figure 2E). The binding affinity was not influenced by the addition of S1P versus vehicle control (MeOH) (*K*_D__MeOH_∼192 nM; *K*_D__S1P_∼202 nM; Figure 2F). For a mapping of binding sites, individual extracellular domains (N-terminus and extracellular loops [ECLs]) of S1PR2 were synthesized as peptides and analyzed for binding to Nogo-A-Δ20 by microscale thermophoresis (Figure 2G). Nogo-A-Δ20 was found to bind primarily to ECL2 (*K*_D_∼280 nM) and 3 (*K*_D_∼350 nM), less strongly to ECL1 (*K*_D_∼2 µM) and negligibly to the N-terminus of S1PR2 (*K*_D_∼11 µM) (Figure 2G). Importantly, binding analysis of the other bioactive domain of Nogo-A, Nogo-66, to S1PR2 extracellular domains revealed only unspecific binding in the high micromolar range (*K*_D ECL1_∼46 µM; *K*_D ECL2_∼7 µM; *K*_D ECL3_∼67 µM) or complete absence of binding (N-terminus) (Figure 2H). Collectively, these data show that Nogo-A-Δ20 but not Nogo-66 binds to specific extracellular domains of the GPCR S1PR2.

### S1PR2 Is Internalized upon Nogo-A-Δ20 Binding

**Figure 3:**
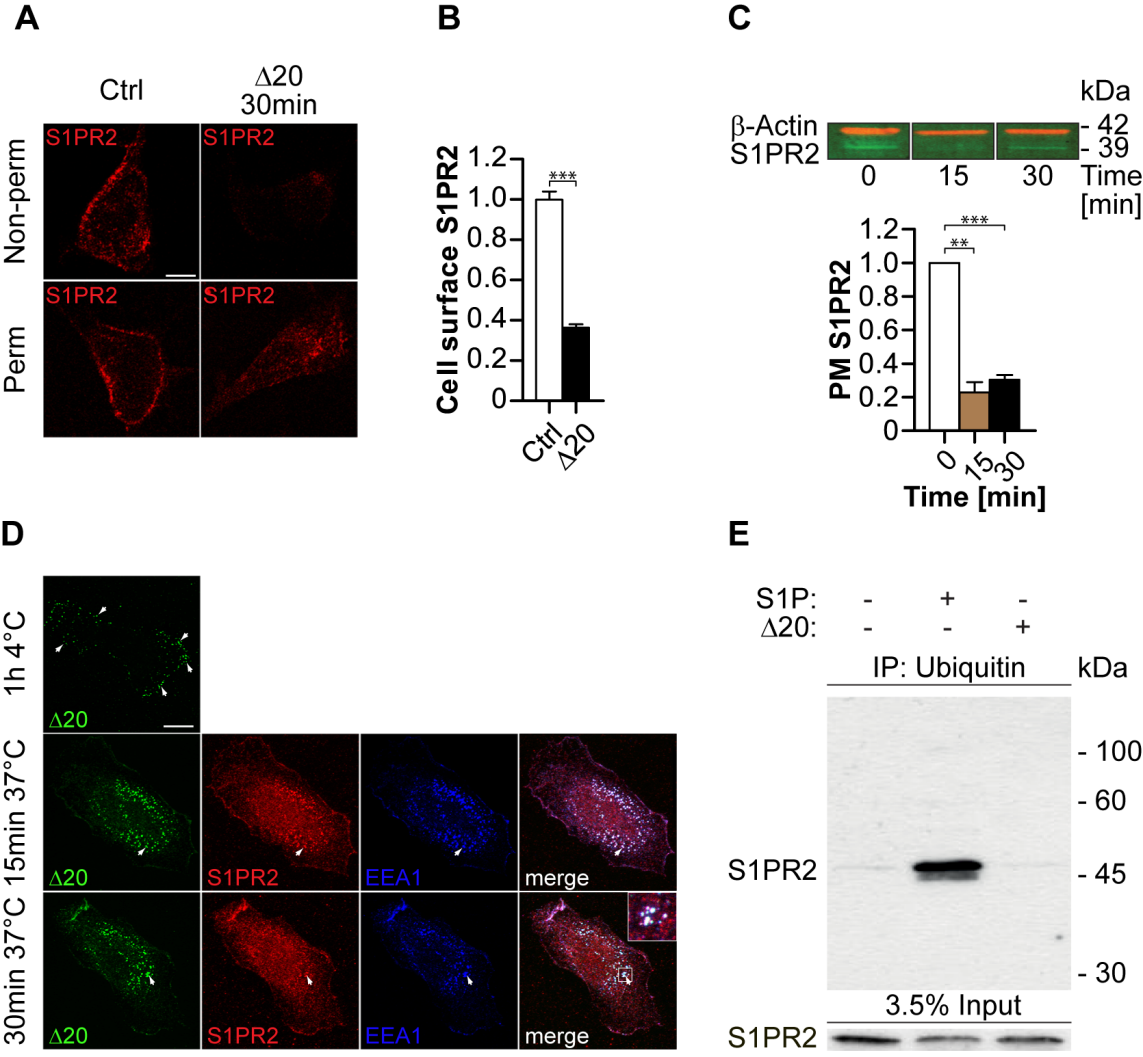
S1PR2 is internalized upon Nogo-A-Δ20 binding. (A) Representative confocal micrographs of 3T3 cells stained alive (Non-perm) or fixed (Perm) for S1PR2 before (control) and 30 min after Δ20 treatment at 37°C. (B) Mean fluorescence intensity quantification of the cell surface staining shown in (A). (C) Addition of Δ20 downregulates cell surface S1PR2 in 3T3 plasma membranes (PM): immunoblot and relative quantification thereof. Loading control: β-Actin. (D) Representative confocal micrographs of 3T3 cells incubated with 1 µM HA-tagged Δ20 for 1 h at 4°C (pulse), which were then subsequently chased for 15 and 30 min at 37°C. Cells were stained with an anti-HA (Δ20), S1PR2, or EEA1 antibody (early endosomes). Arrows indicate cell surface-bound Δ20 (top panel) or colocalization of Δ20 and S1PR2 in early endosomes (middle and bottom panel). The inset panel shows an enlarged view of the boxed region. (E) Western blot analysis of ubiquitinated and non-ubiquitinated protein fractions of 3T3 cells 30 min after Δ20 or S1P treatment. Data shown are means ± SEM (*n* = 3–6 experiments; **p<0.01, ***p<0.001). Scale bars: (A,D) 50 µm.

We have shown previously that Nogo-A-Δ20 is internalized into signaling endosomes upon binding, which results in RhoA activation and growth cone collapse [22]. To investigate whether S1PR2 is co-internalized upon Nogo-A-Δ20 treatment, cell surface S1PR2 expression was analyzed by immunofluorescence using a custom-made antibody (Figures 3A, S2B, and S2C). Cell surface S1PR2 levels were reduced by ∼64% (*p<*0.001) 30 min after addition of Nogo-A-Δ20 (Figure 3B). To confirm this, plasma membranes of 3T3 cells were prepared 15 and 30 min post-incubation with Nogo-A-Δ20 and analyzed for S1PR2 levels by immunoblotting (Figures 3C and S2A). We found that cell surface S1PR2 levels were reduced by ∼77% (*p<*0.01) and ∼70% (*p<*0.001) after 15 and 30 min incubation with Nogo-A-Δ20, respectively, indicating that S1PR2 is internalized upon binding to Nogo-A-Δ20 (Figure 3C). Pulse-chase experiments revealed that the majority of internalized Nogo-A-Δ20 puncta colocalize with S1PR2 as well as with the endosomal marker EEA1 at 15 and 30 min post-incubation with Nogo-A-Δ20 (Figure 3D). Ubiquitination of GPCRs is a critical post-translational modification, which is often dispensable for initial receptor endocytosis but important for endosomal trafficking to proteasome/lysosomal degradation pathways [23],[24]. S1P has been shown to cause S1PR1 monoubiquitination and, in higher concentrations, polyubiquitination, resulting in subsequent GPCR recycling to the membrane or complete degradation, respectively [25]. S1PR2-ubiquitin conjugates were not detected upon internalization of Nogo-A-Δ20 as opposed to S1P (Figure 3E), indicating that Nogo-A-Δ20 signaling is not permanently terminated in the lysosomal degradation pathway [23]–[25]. These results suggest that S1PR2 is rapidly co-internalized with Nogo-A-Δ20 into early endosomes upon binding, which is known to be a key step for Nogo-A-Δ20-mediated growth inhibition [22].

### S1PR2 Mediates Nogo-A-Δ20-Induced Inhibition of Cell Spreading and Neurite Outgrowth

**Figure 4:**
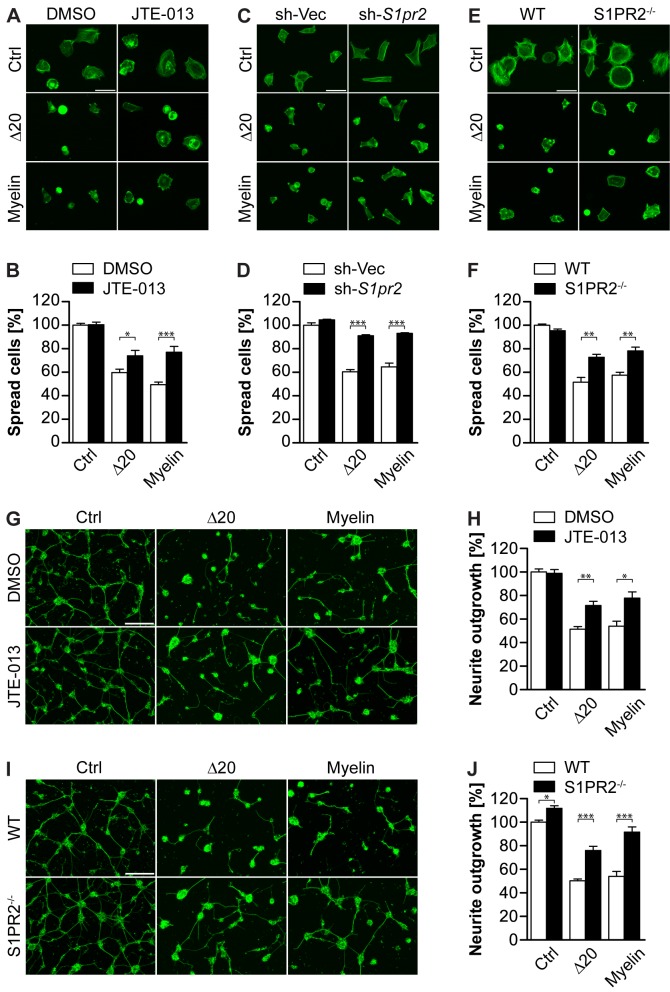
S1PR2 mediates Nogo-A-Δ20- and myelin-induced inhibition of cell spreading and neurite outgrowth. (A,C) Representative pictures of 3T3 fibroblasts treated with JTE-013 or vehicle (DMSO) (A), or stably carrying a *S1pr2* shRNA (sh-*S1pr2*) or empty vector (sh-Vec) construct (C) and plated on control, Nogo-A-Δ20 or myelin substrates. (B,D) Cell spreading quantification of (A) and (C). (E) Representative pictures of MEFs isolated from WT or S1PR2^−/−^ mice and plated on control, Nogo-A- Δ20, or myelin substrates. (F) Cell spreading quantification of (E). Cells were stained with Alexa488-conjugated Phalloidin in (A, C, and E). (G,I) Representative pictures of P5–8 cerebellar granule neurons treated with JTE-013 or DMSO (G), or isolated from S1PR2^−/−^ or WT mice (I) and plated on PLL (ctrl), Nogo-A-Δ20 or myelin substrates. (H,J) Normalized mean neurite length per cell quantification of (G) and (I). Neurons were stained with βIII-Tubulin in (G) and (I). Data shown are means ± SEM (*n* = 3–6 experiments; *p<0.05, **p<0.01, ***p<0.001). Scale bars: 50 µM.

Nogo-A-Δ20 exerts strong inhibitory effects on growth and adhesion of different neuronal cell types and, unlike Nogo-66, also on non-neuronal cells such as 3T3 fibroblasts, which are devoid of NgR1 expression [12]. To determine the functional role of S1PR2 for Nogo-A-Δ20-mediated effects *in vitro*, the well-characterized S1PR2 blocker JTE-013 [26] was tested for its ability to reverse Nogo-A-Δ20-mediated inhibition of cell spreading. Treatment of 3T3 cells with JTE-013 significantly counteracted Nogo-A-Δ20-mediated cell spreading inhibition, resulting in an ∼24% increase of spread cells when compared to vehicle (DMSO) (*p<*0.05) (Figure 4A and 4B). Similarly, on myelin, cell spreading was increased by ∼56% (*p<*0.001) (Figure 4A and 4B). These effects were dose-dependent (Figure S3A) and S1PR subtype-specific (Figure S3B): blockade of S1PR1 with W146, S1PR1 and 3 with VPC-23019, S1PR1, 3, 4, and 5 with FTY-720 or S1PR5 with a function-blocking antibody [27] had no effect on Nogo-A-Δ20-mediated cell spreading inhibition (Figure S3B). In addition, no synergistic effect was observed by combining JTE-013 with any of these blocking agents (Figure S3C), suggesting that solely S1PR2 is responsible for Nogo-A-Δ20-mediated effects in 3T3 cells. To underline the functional importance of S1PR2, its expression was retrovirally silenced in 3T3 cells (sh-*S1pr2*; Figure S4A and S4B). Knockdown of S1PR2 resulted in a very strong increase of cell spreading on a Nogo-A-Δ20 (∼51%; *p<*0.001) or myelin (∼44%; *p<*0.001) substrate when compared to the control vector (sh-Vec) (Figure 4C and 4D). Similarly, primary mouse embryonic fibroblasts (MEFs) isolated from S1PR2^−/−^ mice [28] were significantly less inhibited by Nogo-A-Δ20 (∼41%; *p<*0.01) or myelin (∼36%; *p<*0.01) when compared to wild-type (WT) MEFs (Figure 4E and 4F).

To investigate the functional importance of S1PR2 in Nogo-A-Δ20-mediated neurite outgrowth inhibition, we focused on postnatal day (P) 5–8 cerebellar granule neurons that express S1PR2 (Figure S1B). Pharmacological blockade of S1PR2 using JTE-013 led to a ∼39% (*p<*0.01) and ∼44% (*p<*0.05) increase in outgrowth on a Nogo-A-Δ20 and myelin substrate, respectively (Figure 4G and 4H). Similarly, knockout of S1PR2 also increased neurite outgrowth by ∼51% (*p<*0.001) and ∼69% (*p<*0.001) on a Nogo-A-Δ20 and myelin substrate, respectively (Figure 4I and 4J). Together, these results provide strong evidence that S1PR2 acts as a functional receptor for Nogo-A-Δ20. Importantly, application of JTE-013 had no effect on a growth-inhibitory Nogo-66 or Aggrecan substrate (Figure S5).

### Nogo-A-Δ20 Signals through G_13_, LARG, and RhoA

**Figure 5:**
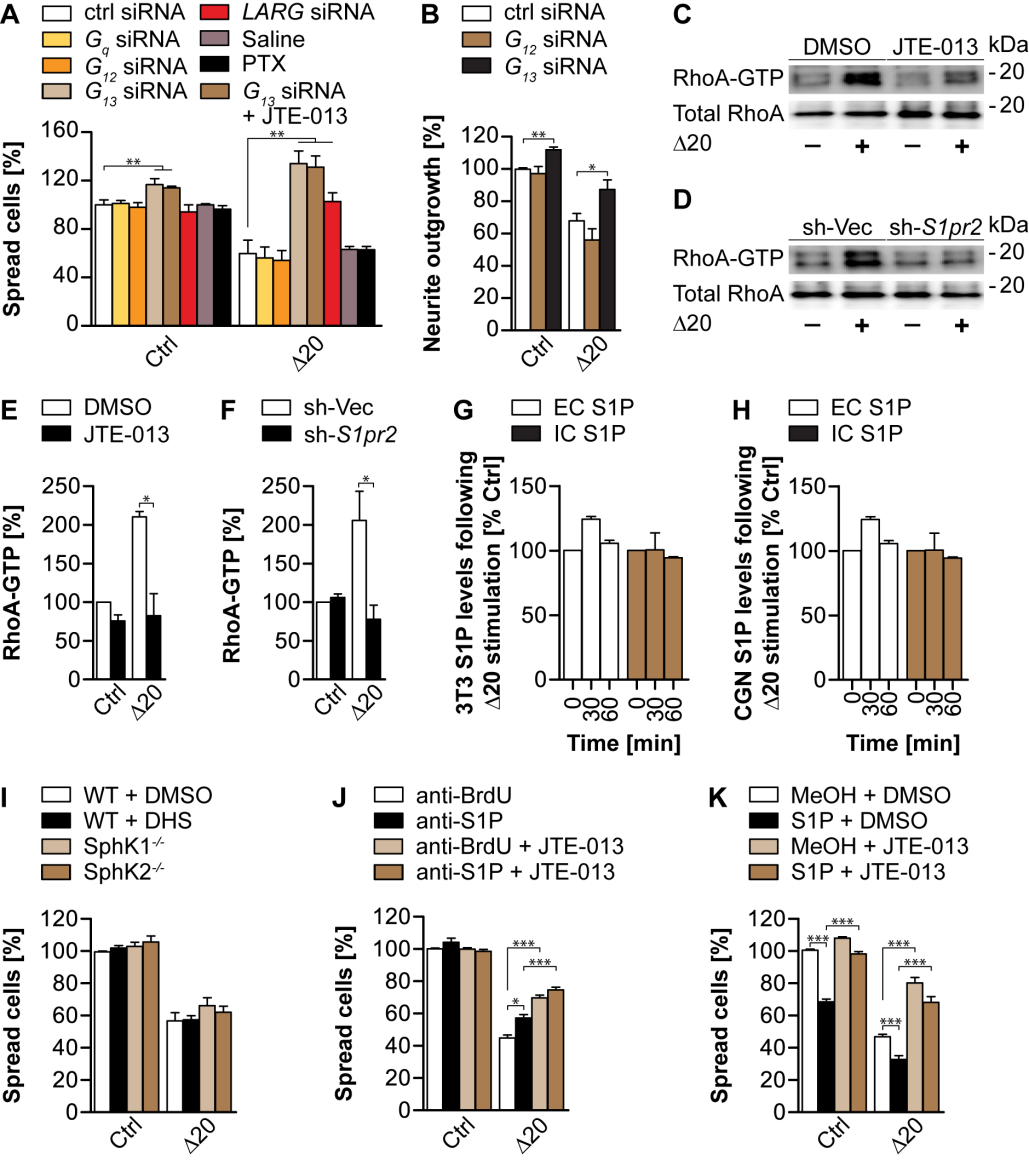
Nogo-A-Δ20 inhibition is mediated via the G_13_-LARG-RhoA signaling axis and can be modulated by exogenous S1P. (A) 3T3 cells transfected with siRNAs against *G_12_*, *G_13_*, *G_q_*, or *Larg*, or control (ctrl) siRNA were replated on a Nogo-A-Δ20 substrate and assessed for cell spreading. G_i/o_ was blocked with Pertussis Toxin (PTX) for which saline was used as control. JTE-013 was co-applied to *G_13_*-siRNA-treated cells to investigate a cumulative effect. (B) Transfection of DIV4 E19 cortical neurons with siRNA against *G_13_* but not *G_12_* similarly rescued Nogo-A-Δ20-induced neurite outgrowth inhibition. (C,D) Nogo-A-Δ20-induced RhoA activation was assessed in JTE-013- versus DMSO-treated cells (C) or in cells carrying a stable knockdown of S1PR2 (sh-*S1pr2*) versus control vector (sh-Vec) (D). (E,F) Relative quantification of (C) and (D), respectively. (G,H) Competitive ELISA quantifications of extra- (EC) and intracellular (IC) S1P levels in 3T3 cells (G) and cerebellar granule neurons (H) before and after 30 and 60 min incubation with Nogo-A-Δ20. (I) Quantification of Nogo-A-Δ20-mediated cell spreading inhibition in the presence of the SphK-specific blocker D,L-*threo*-dihydrosphingosine (DHS) or in SphK1^−/−^ or SphK2^−/−^ MEFs. (J,K) 3T3 cells were plated on a Nogo-A-Δ20 substrate in the presence of the function blocking anti-S1P antibody Sphingomab (J) or of exogenous S1P (K) and assessed for cell spreading. Co-application of JTE-013 significantly reversed the modulatory effects obtained by S1P (K) but not anti-S1P (J). Anti-BrdU antibody or methanol was used as control in (J) and (K). Data shown are means ± SEM (*n* = 3–6 experiments; *p<0.05, **p<0.01, ***p<0.001).

The G proteins G_q_, G_12_, and G_13_ were shown to interact with S1PR2 and to activate the small GTPase RhoA [16],[29]. To determine whether G_q_, G_12_, or G_13_ are implicated in Nogo-A-Δ20-mediated cell spreading inhibition, we transfected small interfering RNAs (siRNAs) targeting the mRNAs of the G proteins (Figure S4C and S4D). Downregulation of G_13_ but not of G_q_ or G_12_ fully rescued cell spreading from ∼63% to ∼134% on Nogo-A-Δ20 when compared to the siRNA control (*p<*0.01) (Figure 5A). No cumulative effect was observed by co-application of JTE-013, suggesting that G_13_ is a key regulator of Nogo-A-Δ20-mediated effects downstream of S1PR2 (Figure 5A). Accordingly, inhibition of the Rac1-coupled G_i/o_ protein [16] with Pertussis toxin (PTX) did not have any effect on Nogo-A-Δ20-mediated cell spreading inhibition (Figure 5A). To assess whether G_13_ is also involved in Nogo-A-Δ20-mediated inhibition of neurite outgrowth, G_13_ was silenced in E19 rat cortical neurons using specific siRNAs (Figure S4E and S4F). Knockdown of G_13_ but not of G_12_ specifically rescued outgrowth from ∼68% to ∼87% on Nogo-A-Δ20 when compared to the siRNA control (*p<*0.05) (Figure 5B). Taken together, these results demonstrate that G_13_ is required for Nogo-A-Δ20-mediated inhibition of cell spreading and neurite outgrowth *in vitro*.

S1PR2 has been shown to couple via G_12/13_ to the RhoGEF LARG to mediate various RhoA-dependent cellular effects [30]. siRNA-mediated downregulation of LARG fully rescued cell spreading from ∼63% to ∼103% on Nogo-A-Δ20 when compared to the siRNA control (*p<*0.01) (Figures 5A, S4C, and S4G). This is in line with LARG-mediated activation of RhoA reported for other repulsive cues such as S1P (via S1PR2 [30]), semaphorin4D (via PlexinB1 [31]), and repulsive guidance molecule RGMa (via Unc5b [32]).

To test whether Nogo-A-Δ20-induced activation of RhoA [22],[33] is S1PR2-dependent, endogenous RhoA activity was measured upon blockade or silencing of S1PR2 in 3T3 cells (Figure 5C–5F). Under control conditions, a ∼2-fold increase in RhoA activation was observed after 20 min of incubation with Nogo-A-Δ20 (Figure 5C and 5D). Upon application of JTE-013 (Figure 5C and 5E) or silencing of S1PR2 (Figure 5D and 5F), RhoA activation was fully suppressed (*p<*0.05). These results suggest that S1PR2 is required for Nogo-A-Δ20-induced RhoA activation, most probably *via* a G_13_-LARG signaling pathway.

### Nogo-A-Δ20-Mediated Inhibition Is Modulated by Exogenous S1P

To determine possible functional interactions of Nogo-A-Δ20 and S1P at the level of S1PR2, we first investigated whether Nogo-A-Δ20 itself modulates S1P production. Extra- (EC) and intracellular (IC) S1P levels were quantified in 3T3 and cerebellar granule neuron cultures after a 30 and 60 min stimulation with Nogo-A-Δ20 (Figure 5G and 5H). No significant changes compared to control levels were detected, indicating that Nogo-A-Δ20 had no influence on S1P production under our experimental conditions (Figure 5G and 5H).

We then addressed the role of endogenous S1P in Nogo-A-Δ20-mediated inhibitory effects. Pharmacological blockade of the S1P-producing enzymes sphingosine kinase (SphK) 1 and 2 using D,L-*threo*-dihydrosphingosine (DHS) [34],[35] had no effect on Nogo-A-Δ20-mediated inhibition of cell spreading, suggesting that SphKs are not downstream elements of Nogo-A-Δ20-induced inhibition (Figure 5I). To confirm this result, MEFs isolated from SphK1^−/−^ or SphK2^−/−^ mice [36] were plated on a Nogo-A-Δ20 substrate. Similarly to SphK blockade, no differences in cell spreading inhibition were observed (Figure 5I).

Because S1P is found in fetal bovine serum (FBS)-containing medium [37] used in our experimental conditions, we investigated if serum-derived S1P modulates Nogo-A-Δ20-mediated inhibition. For this purpose, extracellular S1P was scavenged using the monoclonal anti-S1P antibody Sphingomab [38]. Cell spreading analysis revealed that Nogo-A-Δ20-induced inhibition was alleviated by ∼28% (*p<*0.05) in the presence of the anti-S1P antibody when compared to the anti-BrdU control (Figure 5J). To exclude that disinhibition of Nogo-A-Δ20 signaling by blocking or silencing S1PR2 is mediated by an increased activation of Rac1-coupled S1PR1 through serum-derived S1P, anti-S1P was applied together with JTE-013. No differences could be observed between anti-S1P- and anti-BrdU-treated cells in the presence of JTE-013 (Figure 5J). Together, these results suggest that S1PR2-mediated inhibition by Nogo-A-Δ20 occurs independently of S1P but that S1P can modulate Nogo-A-Δ20-mediated effects. Indeed, addition of S1P to cells resulted in an ∼31% (*p<*0.001) and ∼28% (*p*<0.001) decrease in cell spreading inhibition on a control and Nogo-A-Δ20 substrate, respectively, when compared to the MeOH + DMSO control (Figure 5K). These results point to a modulatory function of S1P in Nogo-A-Δ20-mediated inhibition of cell spreading, presumably by independently activating RhoA-coupled cell surface S1PRs, e.g., S1PR2. Concordantly, S1P has been previously described to modulate cell adhesion and growth of different cell types [18],[27],[39]. To test this hypothesis, JTE-013 was co-applied with S1P. S1P-induced inhibition of cell spreading could be significantly reversed on a control and Nogo-A-Δ20 substrate in the presence of JTE-013 (*p*<0.001) (Figure 5K). Together, these results indicate that S1P can modulate Nogo-A-Δ20-mediated cell spreading inhibition via S1PR2. However, they also suggest that Nogo-A-Δ20 acts independently of SphK or S1P.

### Nogo-A Restricts Long-Term Potentiation via S1PR2 in the Hippocampus and Motor Cortex

**Figure 6:**
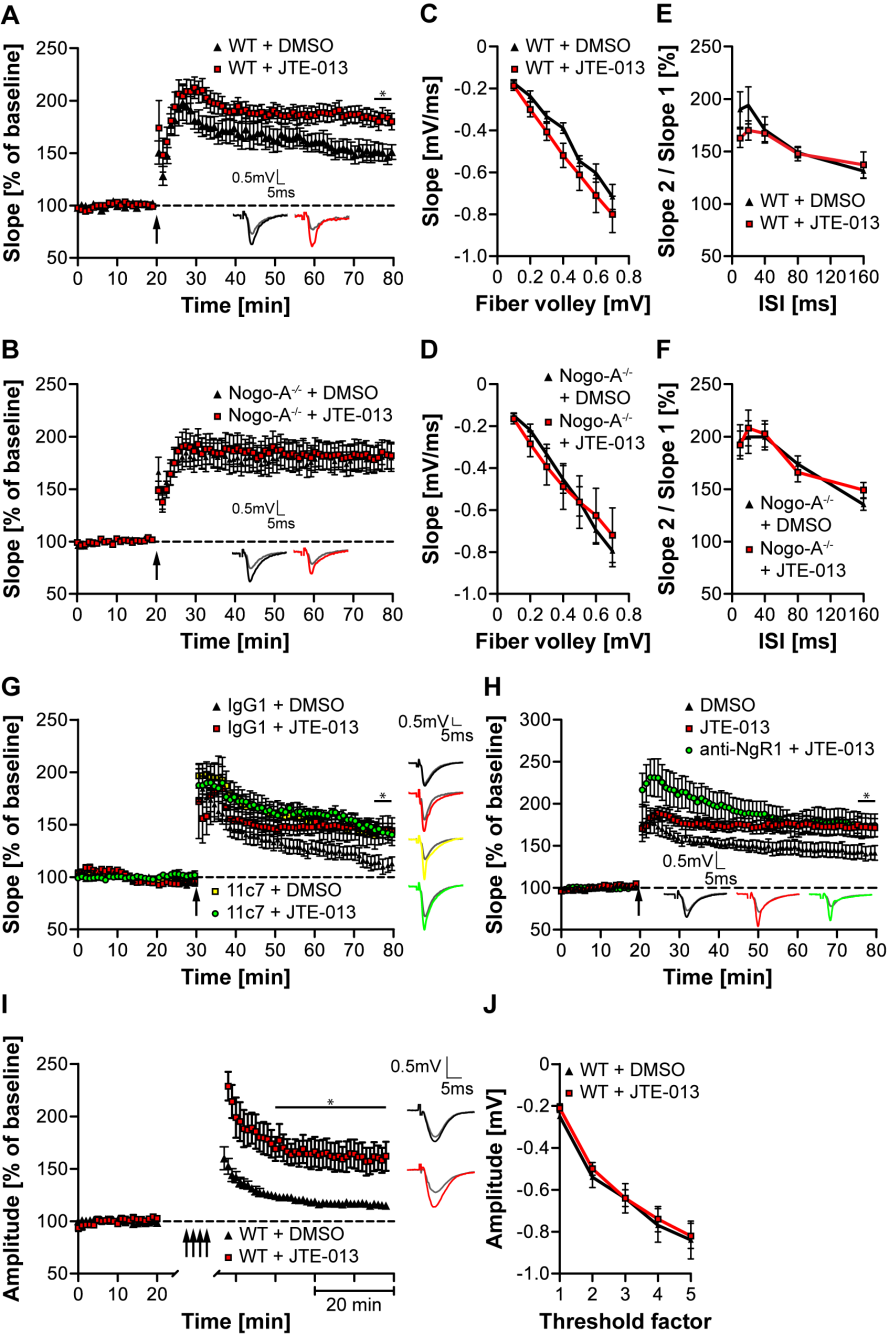
Blockade of S1PR2 phenocopies the increase in hippocampal and cortical LTP observed upon Nogo-A neutralization. (A,B) Hippocampal WT (A) and Nogo-A^−/−^ (B) slices were treated with JTE-013 or vehicle (DMSO) (WT_DMSO_: *n* = 8; Nogo-A^−/−^_DMSO_: *n* = 10; WT_JTE-013_: *n* = 11; Nogo-A^−/−^_JTE-013_: *n* = 9). 60 min after theta-burst stimulation (arrow), a significant difference in LTP could be observed between JTE-013 and DMSO treatment in WT (A) but not Nogo-A^−/−^ (B) slices. (C,D) Input-output strength revealed no differences in JTE-013- versus DMSO-treated slices of WT (C) and Nogo-A^−/−^ (D) mice (WT_DMSO_: *n* = 6; Nogo-A^−/−^_DMSO_: *n* = 6; WT_JTE-013_: *n* = 7; Nogo-A^−/−^_JTE-013_: *n* = 6). (E,F) PPF revealed no alterations in JTE-013- versus DMSO-treated slices of WT (E) and Nogo-A^−/−^ (F) mice (WT_DMSO_: *n* = 7; Nogo-A^−/−^_DMSO_: *n* = 6; WT_JTE-013_: *n* = 5; Nogo-A^−/−^_JTE-013_: *n* = 6). (G) LTP was measured upon simultaneous neutralization of S1PR2 using JTE-013 and of Nogo-A using 11c7 (IgG1 + DMSO: *n* = 7; IgG1 + JTE-013: *n* = 6; 11c7 + DMSO: *n* = 8; 11c7 + JTE-013: *n* = 6). (H) LTP was measured upon simultaneous neutralization of S1PR2 using JTE-013 and of NgR1 using anti-NgR1 (DMSO: *n* = 7; JTE-013: *n* = 9; anti-NgR1 + JTE-013: *n* = 8). (I) Rat motor forelimb area brain slices were treated with JTE-013 (*n* = 7) or DMSO (*n* = 8). Peak amplitudes were significantly larger in JTE-013- versus DMSO-treated slices upon repeated inductions of LTP (multiple arrows). (J) Input-output strength revealed no differences in JTE-013- (*n* = 8) versus DMSO-treated (*n* = 12) cortical slices. Insets show representative traces. Data shown are means ± SEM (*p<0.05). *n* indicates the number of mice used.

Growing evidence suggests that Nogo-A plays an important role in restricting synaptic plasticity [6],[9],[11]. S1PR2 is expressed in CA1 and CA3 pyramidal neurons (Figure 1A and 1B). In order to investigate the role of the Nogo-A/S1PR2 axis in long-term potentiation (LTP), hippocampal slices of WT and Nogo-A^−/−^ mice were tested for LTP after acute blockade of S1PR2 using JTE-013. In WT slices, application of JTE-013 resulted in a significant increase in LTP compared with vehicle (DMSO) (∼22%; *p<*0.05) (Figure 6A). In contrast, no differences in LTP were detected in Nogo-A^−/−^ slices treated with JTE-013 or vehicle, suggesting that Nogo-A is required for S1PR2-mediated effects on LTP (Figure 6B). No differences in input-output (I/O) curves and paired-pulse facilitation (PPF) could be observed by application of JTE-013, suggesting that S1PR2 blockade does not alter baseline synaptic transmission or the properties of presynaptic terminals (Figure 6C–6F). In order to confirm the specificity of S1PR2, LTP was measured after blockade of the remaining S1PRs (Figure S6A and S6B). No differences in LTP and PPF could be observed upon application of VPC-23019 or FTY-720, emphasizing the specificity of a functional Nogo-A/S1PR2 interaction (Figure S6A–S6C). Next, we investigated LTP, baseline synaptic transmission as well as PPF in S1PR2^−/−^ versus WT hippocampal slices. No significant changes in LTP, I/O, or PPF could be observed in S1PR2^−/−^ versus WT mice (Figure S6D–S6F) as opposed to acute neutralization of S1PR2. These results mirror those obtained in Nogo-A KO [7] or NgR1 KO [8] mice and suggest that there is a strong drive for genetic compensation in this functionally very important system. [11].

Next, the outcome of a combined neutralization of the ligand Nogo-A by the function-blocking anti-Nogo-A antibody 11c7 [12] and of the receptor S1PR2 by JTE-013 was analyzed. A synergistic effect of the combined treatment as compared to either treatment alone would indicate that additional molecules, e.g., S1P are involved in S1PR2-mediated LTP restriction. A similar increase in LTP for all treated groups when compared to the IgG1 + DMSO control with no difference between the groups was observed (Figure 6G). To assess the relative contribution of the Nogo-A receptors NgR1 and S1PR2 onto Nogo-A-mediated restriction of synaptic plasticity, we simultaneously blocked both receptors. No significant difference could be observed between application of JTE-013 alone versus the combined application of JTE-013 and of the function-blocking anti-NgR1 antibody (Figure 6H).

Finally, we investigated the effect of S1PR2 blockade on long-term depression (LTD) in the hippocampus. In line with the results obtained after acute Nogo-A neutralization [7], JTE-013 application did neither modulate LTD induction nor maintenance compared with control conditions (Figure S6G).

Recent data indicate that Nogo-A also restricts synaptic plasticity in the primary motor cortex [11]. LTP saturation in this region was also significantly increased in JTE-013 versus DMSO-treated slices (∼39%; *p<*0.001) (Figure 6I). No differences in the I/O curves were observed after S1PR2 blockade, indicating that the JTE-013-mediated increase in synaptic plasticity was not due to alterations in baseline synaptic transmission (Figure 6J). Together, these results show that Nogo-A represses synaptic plasticity in the hippocampus and motor cortex via S1PR2.

## Discussion

Two distinct domains of Nogo-A can induce growth inhibition: Nogo-A-Δ20 and Nogo-66. Here, we identified the GPCR S1PR2 as the first functional receptor for the inhibitory Δ20 domain of Nogo-A. S1PR2 fulfills essential key criteria to be a Nogo-A-Δ20-specific receptor: (i) Expression in the CNS as well as in non-neuronal Nogo-A-Δ20-responsive cells; (ii) high-affinity binding to Nogo-A-Δ20; (iii) prerequisite for Nogo-A-Δ20-induced inhibition of cell spreading and neurite outgrowth; (iv) Nogo-A-Δ20-induced activation of RhoA; (v) restriction of hippocampal and cortical synaptic plasticity.

### S1PR2 Is a Receptor for a Lipid and a Protein Ligand

Until very recently, GPCRs were generally thought to be activated by physical and low MW chemical stimuli [40]. However, a few adhesion GPCRs were found to also bind to membrane-bound and matrix ligands via an extended N-terminal region [41],[42]. Many of these receptors such as EGF-containing CD97, the first GPCR shown to bind to the cellular ligand decay accelerating factor, are predominantly expressed by immune cells [43]. To our knowledge, Nogo-A is the first mammalian membrane protein shown to bind to and signal through a non-orphan GPCR of the rhodopsin-like family. In contrast to adhesion GPCRs, S1PR2 does not bind Nogo-A-Δ20 via its N-terminal domain.

The recent characterization of the crystal structure of S1PR1 provided substantial structural information on its activation by S1P [19]. Access of the ligand to the binding pocket from the extracellular space is occluded by the N-terminus and the ECLs, and may be gained from within the membrane [19]. Our data provide strong evidence that Nogo-A-Δ20 primarily interacts with ECL2 and ECL3 of S1PR2, suggesting a different mechanism of activation compared to S1P. Our results also suggest that S1PR2-mediated inhibition by Nogo-A-Δ20 does not require S1P but can be exogenously modulated by the latter. Although binding of Nogo-A-Δ20 to S1PR2 does not require S1P, modulation of receptor-specific physiological outputs by binding of the bioactive lipid to its pocket within the membrane may further expand the signaling repertoire of S1PR2. It may also enable fine-tuned cellular responses depending on the ratio of ligands present under given conditions, as recently suggested for the receptor for advanced glycation endproducts (RAGE) [44]. Future biochemical and structural studies will be necessary to address this and show how binding is transferred into ligand-specific G-protein-dependent signaling. Detailed investigations will also need to determine whether the presence of additional receptors, i.e. NgR1, affects the binding properties of Nogo-A-Δ20 to S1PR2 as described for other multi-receptor systems, e.g., the viral surface glycoprotein gp120 to CD4 and the GPCR co-receptor CCR5 [45]. We could show that Nogo-A interacts with S1PR2 *in trans*. However, interaction at the surface of the same cell *in cis* might also be possible, similar to what has been proposed for the Nogo-A–NgR1 interaction in Purkinje cells recently [46]. Yet, such mechanisms have not been proven and their existence needs to be investigated in detail.

### Multi-ligand/Multi-receptor Cross-Talk

The classic “one ligand–one receptor” paradigm has recently been challenged by an increasing number of multi-ligand/multi-receptor interactions, which could be identified in different biological systems, adding another level of complexity for fine-tuning of cellular responses [20]. Examples include neurotrophin receptors, Wnt receptors, and receptors for axonal guidance molecules such as plexins and neuropilins [20]. We propose that the Δ20 domain of Nogo-A binds to S1PR2 and the Nogo-66 loop to NgR1 and/or PirB, resulting in the formation of a multi-site/multi-ligand receptor complex. NgR1 and PirB can also interact with ligands other than Nogo-A, thereby increasing the dynamics of signal transduction [6],[9]. Additional Nogo-A co-receptors and downstream signaling components potentially located within or attached to these multi-receptor complexes might further amplify Nogo-A-mediated inhibitory effects. It was recently demonstrated that canonical GPCR signaling also occurs from endosomes for, e.g., the Wnt receptor Frizzled [47] and the β2-adrenoceptor [48]. Along this line, the Nogo-A-Δ20/S1PR2 complex is co-internalized into endosomes, from which signaling may be sustained. Currently, the concerted action and downstream trafficking of all these receptor components is still poorly understood, in particular *in vivo*. Future studies will need to assess whether all Nogo-A (co-)receptors are found within the same complex or in different membrane microdomains, and how the receptor composition varies between different cell types, developmental stages, and pathophysiological conditions.

### Interfering with Nogo-A/S1PR2 Signaling Increases Synaptic Plasticity

Nogo-A stabilizes neuronal networks by restricting CNS plasticity [2],[9]. Acute neutralization of Nogo-A or NgR1 in hippocampal slices was shown to induce an increase in LTP at CA3-CA1 synapses [7]. On the other hand, conventional knockouts of Nogo-A, PirB, or NgR1 do not show significant modulations in LTP, presumably due to compensatory mechanisms [7],[8],[10],[11],[49]. This is well in line with the lack of LTP modulation observed in S1PR2^−/−^ mice. A novel transgenic rat model in which Nogo-A expression was silenced but not completely ablated by using a synthetic anti-Nogo-A microRNA leaving the genomic locus intact showed a significant increase in LTP in the hippocampus as well as in the motor cortex [11]. This underlines the strong drive for genetic compensation after complete ablation of components within this functionally very important system. Our present findings revealed an increase in hippocampal and cortical LTP when acutely interfering with S1PR2 signaling by JTE-013. Notably, no JTE-013-mediated increase in hippocampal LTP was observed in Nogo-A^−/−^ mice, underlining the plasticity-restricting role of Nogo-A/S1PR2 signaling independently of S1P. Indeed, CA3–CA1 LTP was shown to be independent of SphK/S1P receptor signaling [50]. Interestingly, the blockade of both Nogo-A receptors NgR1 and S1PR2 does not show an additive effect on LTP potentiation, suggesting that both receptor-evoked responses induced by different domains of Nogo-A converge onto the same signaling pathways. However, detailed mechanisms and kinetics by which Nogo-A/S1PR2-NgR1 modify synaptic plasticity remain to be analyzed.

### Conclusion

Our finding that the GPCR S1PR2 binds two structurally unrelated molecules, a low MW sphingolipid and the high MW membrane protein Nogo-A, by distinct sites contributes to and extends the paradigm shift from a classical linear model of GPCR signaling towards a more dynamic model with shared components and intramolecular cross talks [51],[52]. It will be important to understand to which extent S1P affects signaling induced by Nogo-A and vice-versa. Detailed high-resolution structural characterization of the receptor in complex with S1P, Nogo-A, or both will be required to unravel the mechanistic properties of these two signaling systems. Furthermore, the cell-specific interplay of S1PR2 with known receptors and co-receptors for Nogo-A needs to be determined in detail with regard to their corresponding physiological effects. This information will be the basis for the design of novel molecular tools to better understand the roles of Nogo-A/S1PR2 signaling for CNS plasticity and repair.

## Materials and Methods

### Animals

All animal experiments were performed with the approval of and in strict accordance with the guidelines of the Zurich Cantonal Veterinary Office. All efforts were made to minimize animal suffering and to reduce the number of animals required.

*S1pr2* ^−/−^ (B6.129S6-S1pr2^tm1Rlp^) mice were produced by targeted mutagenesis as described previously [28] and backcrossed to C57BL/6 background.

### Ensembl Accession Numbers

Accession numbers mentioned in this paper from the Ensembl Genome Browser (www.ensembl.org) are: *Gna12*, ENSMUSG00000000149; *Gna13*, ENSMUSG00000020611; *Larg*, ENSMUSG00000059495; *RhoA*, ENSMUSG00000007815; *Rtn4*, ENSMUSG00000020458, ENSRNOG00000004621; *S1pr2*, ENSMUSG00000043895.

### Yeast Two-Hybrid Screen

The Nogo-A-Δ20 recombinant protein fused to the activation domain of the GAL4 transcription factor was used as bait to screen for interacting proteins from cDNAs from adult and fetal brain libraries (Clontech) using the yeast two-hybrid (Y2H) method as described previously [53]. Briefly, the cDNA encoding bait fragment was generated by PCR, cloned into pDONR201, and transferred into GATEWAY (Invitrogen)-compatible versions of pGBT9 by the LR reaction. Yeast strain CG1945 (Clontech) was transformed with the resulting vector. cDNA libraries were transformed into Y187 strain (Clontech). Bait- and prey-expressing yeasts were mated in YPDA in the presence of 10% polyethylene glycol 6000. Medium was changed to selective medium (synthetic dextrose) lacking Leu, Trp, and His with the following additives: 0.5% penicillin/streptomycin (50 µg/ml, Invitrogen), 50 µm 4-methylumbelliferyl-α-d-galactoside (Sigma), and varying concentrations of 3-amino-1, 2, 4-triazole (3-AT, Sigma). Different concentrations of 3-AT were tested in pre-screens, varying from 0–60 mM. 60 mM 3-AT produced <20% hits; 130 mM 3-AT was used in the main screen, resulting in ∼0.5% strong bait-prey interactions. Mating efficiency was determined by plating of cells on selective agar plates. The cell suspension was aliquoted into microtiter plates (96 wells/plate, flat bottom, 200 µl/well) and incubated for 3–7 days. Positive clones were screened by determining fluorescence on a SpectraFluor fluorometer (Tecan) at 465 nm (excitation at 360 nm). Wells that displayed fluorescence above background were identified and automatically collected by a Tecan Genesis 200 robot. Selected cells were passaged twice and transferred to an agar plate before PCR amplification of the library inserts. After DNA sequencing and sequence blasting, all bait-prey interactions were assessed for intrinsic prey promiscuities by comparison with in house databases containing prey information on binding frequencies obtained from previous studies [53]. Ingenuity Pathway Analysis (IPA, Ingenuity Systems) was subsequently used to identify if interaction partners signal *via* RhoA.

### Tissue Preparation and Cell Culture

Total myelin protein extracts were prepared from the brains and spinal cords of adult Wistar rats as described previously [12]. Swiss 3T3 (ATCC), NIH 3T3 cells (ATCC), and HEK293T cells (ATCC) were maintained in DMEM containing 10% neonatal calf serum (Invitrogen). Postnatal (P5–8) cerebellar granule neurons were prepared as described previously [12]. Embryonic day (E) 19 rat cortical neurons were prepared as described previously [8]. Primary MEFs were isolated and immortalized as described previously [54]. Each primary fibroblast culture was isolated from a single E9.5 S1pr2^−/−^ or WT littermate mouse.

### siRNA, shRNA, and Recombinant Fusion Proteins

*S1PR2* (ENST00000317726) was PCR-amplified from human blood RNA, cloned into the EcoRI/Xho sites of the pcDNA5 vector (Invitrogen) and fully sequenced. The mouse sequences of the siRNAs used are *G_12_ (Gna12)*: GCGACACCAUCUUCGACAACAU, *G_13_ (Gna13)*: CUGGGUGAGUCUGUAAAGUAUU, *G_q_ (Gnaq)*: GCUGGUGUAUCAGAACAUC, and *Larg*: sc-41801 (Santa Cruz Biotechnology). The rat sequences are *G_12_* ON-TARGETplus siRNA SMARTpool L-088001-02-0005 (Thermo Scientific) and *G_13_* ON-TARGETplus siRNA SMARTpool L-086608-02-0005 (Thermo Scientific). A scrambled siRNA sequence was used as control (Dharmacon). NIH 3T3 cells were transfected using Lipofectamine LTX according to the manufacturer's instructions (Invitrogen). E19 cortical neurons were transfected at days *in vitro* (DIV) 4 using DharmaFECT 3 (Dharmacon) according to the manufacturer's instructions. Quantification of the respective mRNA knockdown was performed by qRT-PCR. Quantification of protein knockdown was performed by FACS analysis.

Silencing of *S1pr2* by retroviral transduction of shRNA constructs was done by using phoenix helper-free retrovirus producer lines with pSIR delta HRCG U6 for the generation of helper-free retroviruses as described below [55]. The following shRNA construct targeting *S1pr2* mRNA transcript was used: ACCAAGGAGACGCTGGACATG [56]. Empty vector was used as control. Quantification of the respective mRNA knockdown was performed by qRT-PCR. Quantification of protein knockdown was performed by FACS analysis.

Recombinant protein Nogo-A-Δ20 (rat aa544-725) was purified as described previously [12]. Briefly, BL21/DE3 *Escherichia coli* were transformed with the pET28 expression vector (Novagen) containing His-/T7- or His-/HA-tagged Nogo-A-Δ20 and cultured at 37°C to reach an OD of 0.6 AU. Protein expression was induced by addition of 1 M IPTG for 2 h at 30°C. Fusion proteins were purified using Co^2+^-Talon Metal Affinity Resin (Takara Bio Inc.). Nogo-A-ext (rat aa1–979) was cloned into the KpnI and XhoI restriction sites of the pEXPR-IBA5 expression vector and the recombinant protein was purified from transiently transfected HEK293T cells using *Strep*-tactin chromatography (IBA).

### qRT-PCR

RNA was isolated with RNeasy Micro kit (Qiagen). For synthesis of cDNA we used SuperScript III reverse transcriptase (Invitrogen). qRT-PCR was done as described before using the LightCycler 480 System (Roche, [57]). To determine the relative expression of the target genes *Gna12*, *Gna13*, *Gnaq*, *Larg*, and *S1pr2* we used *Tubb1* and *Eef1a1* as housekeeping genes. The following primers were used: Gna12_FWD: 5′-CATGCGATGCTGCTAAGCTCAC-3′, Gna12_REV: 5′-TGTGTGTTCACTCTGGGAGGTG-3′; Gna13_FWD: 5′-ACTAACCGTGCCTCTTCAATGGC-3′, Gna13_REV: 5′-AGGCACCCAACAAGAACACACTG-3′; Gnaq_FWD: 5′-TGGGGACAGGGGAGAG-3′, Gnaq_REV: 5′-TGGATTCTCAAAAGCAGACAC-3′; S1pr2_FWD: 5′-CACAGCCAACAGTCTCCAAA-3′, S1pr2_REV: 5′-TGTTCCAGAACCTTCTCAGGA-3′; Larg_FWD: 5′-GAATCATCAAGGTGAATGG-3′, Larg_REV: 5′-CTGGTGATTCTCTCCATATTC-3′; Tubb1_FWD: 5′-GCAGTGCGGCAACCAGAT-3′, Tubb1_REV: 5′-AGTGGGATCAATGCCATGCT-3′; Eef1a1_FWD: 5′-TCCACTTGGTCGCTTTGCT-3′, Eef1a1_REV: 5′-CTTCTTGTCCACAGCTTTGATGA-3′.

All samples were analyzed in triplicates. Melting curve analysis of PCR products followed by gel electrophoresis was performed to verify amplicons.

### Antibodies and Pharmacological Blockers

The following primary antibodies were used: β Tubulin (Chemicon, MAB3408; 1∶1,000), βIII Tubulin (Promega, G712A; 1∶1,000), β-Actin (Sigma, A5441; 1∶1,000), BrdU (AbD Serotec, function-blocking experiments: 5 µg/ml), DAPI (Invitrogen, D1306, 1∶1,000), EEA1 (Cell Signaling, 2411; 1∶100), GAPDH (Abcam, ab8245; 1∶20,000), HA (Roche, 11867423001, 1∶200), His (Santa Cruz, sc-804, 1∶500), Pan-CDH (Abcam, ab6528; 1∶1,000), Nogo-A (1∶10,000, [58]), Nogo-A (Rb173A/Laura, 1∶200), Nogo-A/B (Bianca, Rb1, 1: 20,000, [12]), Phalloidin-Alexa488 (Invitrogen; 1∶500), RhoA (Cell Signaling, 2117; 1∶1,000), S1PR2 (Imgenex, IMG-6135A; 1∶250), S1PR2 (AbD Serotec custom made HuCAL antibody AbD14533.1 addressing extracellular S1PR2 ECL2; WB 1∶1,000; IHC 1∶100; TEM 1∶100), S1PR2 (Santa Cruz, sc-365589; 1∶500), S1PR5 (Abcam, ab13130; 1∶500; function-blocking experiments: 5 µg/ml), sphingosine 1-phosphate (Funakoshi, 274594052; function-blocking experiments: 5 µg/ml), Ubiquitin (Enzo Life Sciences, UWO150; 1∶1,000).

The following secondary antibodies were used: Alexa488-conjugated goat anti-mouse IgG (Invitrogen; 1∶1,000), Alexa488-conjugated goat anti-rabbit IgG (Invitrogen; 1∶1,000), Alexa488-conjugated goat anti-rat IgG (Invitrogen; 1∶1,000), Biotin SP-conjugated AffiniPure goat anti-rabbit IgG (Jackson ImmunoResearch Laboratories; 1∶250), Biotin SP-conjugated AffiniPure goat anti-human IgG F(ab′)_2_ fragment specific (Jackson ImmunoResearch Laboratories; 1∶250), Cy3-conjugated Streptavidin (Jackson ImmunoResearch Laboratories; 1∶500), Cy5 goat anti-rabbit (Invitrogen; 1∶500), FITC-conjugated goat anti-human IgG (Fab specific; AbD Serotec), HRP-conjugated goat anti-human IgG (Fab specific; AbD Serotec), HRP-conjugated goat anti-rabbit IgG (Fab specific; Amersham), HRP-conjugated goat anti-mouse IgG (Fab specific; Amersham),

The following pharmacological blockers used in this study have been dissolved according to the manufacturer's instructions: W146 (Avanti Polar Lipids), VPC-23019 (Avanti Polar Lipids), JTE-013 (Tocris Bioscience), FTY-720 (Cayman Chemical), and DHS (Enzo Life Sciences). Nogo-66 was purchased from R&D Systems. Sphingosine 1-phosphate and Aggrecan were purchased from Sigma.

### Binding Assays

Immobilization-based binding assays were performed on an Octet Red Instrument (*forté*BIO). Recombinant S1PR2 and control membrane preparations (Millipore) were immobilized on amine-reactive biosensors (25 µg/ml; *forté*O) in HBSN running buffer (BIAcore) supplemented with 10 mM MgCl_2_. Nogo-A-ext protein was serially diluted and allowed to bind the saturated biosensor tips for 15 min at 1,000 rpm at 30°C. For experiments including S1P, 1 µM S1P was added together with Nogo-A-ext. Methanol was used as vehicle control. The binding response was normalized for baselines differences between runs and binding affinities (*K*_D_) were calculated from a nonlinear fit according to the double-reference subtraction method in GraphPad Prism5 (GraphPad software). Data shown are the average of three to five experiments per condition.

Microscale thermophoresis ligand binding measurements were performed using a Nanotemper Monolith NT.115 (Nano Temper technologies) as previously described [59]–[60]. Briefly, recombinant Nogo-A-Δ20 was fluorescently labeled using the Amine Reactive Protein labeling kit RED (L001, Nano Temper technologies). The N-terminus and individual ECLs of S1PR2 were synthesized as peptides (JPT Peptide Technologies, sequences: N-terminus, MGGLYSEYLNPEKVQEHYNYTKETLDMQETPSRK; ECL1, LSGHVTLSLTPVQW; ECL2, NCLNQLEACSTVLPLYAKHYVL; ECL3, SILLLDSTCPVRACPVLYK; ECL1-scrambled negative control, VGLSQVWTSLPTLH). A constant concentration of Nogo-A-Δ20 (∼40 nM) was incubated with the different serially diluted peptides in PBS containing 0.025% Tween-20 at pH 7.4. 3–5 µl of each sample was loaded into a hydrophilic glass capillary (K004, Nano Temper technologies) and thermophoresis analysis was performed (LED 60%, IR Laser 20%) [59],[60]. MST data were normalized for baseline differences between runs and *K_D_* values were calculated using non-linear regression assuming a Hill coefficient of 1.0 (GraphPad Prism).

Immunoprecipitation was performed with Nogo-A-Δ20 and S1PR2 membrane preparations using the His Protein Interaction Pull-Down kit following the manufacturer's instructions (Pierce). Heat-inactivated Nogo-A-Δ20 was used as control.

Co-immunoprecipitation was done using whole mouse brain tissue from P10 Nogo-A^−/−^, S1PR2^−/−^, and WT mice. Briefly, tissue was lysed with RIPA buffer (50 mM Tris-HCl [pH 7.2], 150 mM NaCl, 0.1% SDS, 0.5% Na.Deoxycholate, 1% NP-40) containing cOmplete Mini EDTA-free protease inhibitor cocktail tablets (Roche). Co-Immunoprecipitation was performed using the Pierce Co-IP Kit (Pierce 26149) according to the manufacturer's instructions.

### *In Vitro* Bioassays

3T3 fibroblast spreading assays and P5-8 cerebellar granule neurons neurite outgrowth assays were performed as described previously [12]. Briefly, four-well plates (Greiner) were coated with 40 pmol/cm^2^ Nogo-A-Δ20 or 5 µg/cm^2^ myelin at 4°C overnight. Nogo-66 Fc was used at a concentration of 500 nM and Aggrecan at 1,000 ng/ml. In outgrowth experiments, wells were precoated with 0.3 µg/ml for 1 h at 37°C before addition of the different substrates. 3T3 cells were plated at 7,000 cells per cm^2^ for 1 h at 37°C and 5% CO_2_, fixed with 4% paraformaldehyde (PFA) and stained with Phalloidin-Alexa-488. Mouse P5-8 cerebellar granule neurons were plated at 7.5×10^4^ cells per cm^2^, cultured for 24 h at 37°C and 5% CO_2_, fixed with 4% PFA and stained with anti-βIII tubulin. Each experiment was performed at least three times in four replicate wells. Spreading was quantified manually in a blinded manner and mean neurite length was quantified using the MetaMorph software (Molecular Devices). The mean neurite length is referred to as the mean total length of all neurites per cell. 3T3 cells were classified as spread cells if they bear at least two lammelipodial processes longer than one cell body diameter. Round cells were classified as non-spread. Data were normalized to baseline and plotted as average ± standard error of the mean (SEM). Cells were imaged with a Leica DM5500B microscope equipped with HCX PL FL Dry 10×/0.3 and 20×/0.5 objectives in a semi-automated way. Statistical analysis was performed in GraphPad Prism5 using a one-way ANOVA test followed by a Bonferroni *post hoc* test or by using an unpaired Student's t-test. All inhibitors were used at a concentration of 100 nM if not elsewhere specified.

### Internalization Assays and Flow Cytometry Analysis

Plasma membranes of 3T3 cells were prepared as described before [61] and after treatment with 1 µM T7-tagged Nogo-A-Δ20. Nogo-A-Δ20 internalization assays were performed as described previously after treatment of 3T3 cells with 1 µM HA-tagged Nogo-A-Δ20 [22]. Briefly, 3T3 cells were incubated with 1 µM Nogo-A-Δ20 for 1 h on ice (pulse) and subsequently chased for 15 and 30 min at 37°C. Flow cytometry-based quantification of S1PR2, G13, and LARG expression on 3T3 cells and CGNs, respectively, was done in a BD FACSCalibur.

### Ubiquitination Assay

3T3 cells were starved in serum-free medium for 24 h. 1 µM S1P or Nogo-A-Δ20, respectively, was added to 3T3 cells for 60 min. Isolation of ubiquitinated protein fractions was done using UbiCapture-Q (Enzo Life Sciences). Finally, western blot analysis was performed to detect S1PR2 and ubiquitin.

### RhoA Pulldown

3T3 cells were serum-starved overnight and treated for 20 min with 1 µM Nogo-A-Δ20 or heat-inactivated Nogo-A-Δ20 control protein. Pulldown of activated RhoA-GTP was subsequently performed using the RhoA Activation Assay Biochem Kit according to the manufacturer's instructions (Cytoskeleton, Inc.).

### S1P Quantification

3T3 cells or CGNs were cultured up to 80%–85% confluence in 15 cm dishes and serum starved for 24 h prior to the experiment. Nogo-A-Δ20 was added to the cells at a concentration of 1 µM. After 15, 30, and 60 min, 3T3 cells and CGNs were lysed in 400 µl lysis buffer (20 mM PIPES, 150 mM NaCl, 1 mM EGTA, 1% v/v Triton X-100, 1.5 mM MgCl_2_, pH 7.4). Lysates were frozen immediately at −80°C. Protein concentration was measured and cell lysates (1∶10 in delipidized human sera) were analyzed with the Echelon S1P ELISA kit according to the manufacturer's instruction. Serum free cell culture medium was directly diluted 1∶10 in delipidized human serum and subsequently analyzed with the S1P ELISA kit.

### Immunohistochemistry

Immunohistochemistry was performed as described previously [62]. Briefly, animals were transcardially perfused with Ringer's solution, followed by 4% PFA. Prior to staining, sections were treated with 0.2% glutaraldehyde and 50 mM Tris-glycine (pH 8.0). After antigen retrieval *via* microwaving three times for 10 s at 600 W, the sections were treated with Kryofix (Merck) for 10 min followed by 0.3% Triton X-100 for 10 min. S1PR2 was detected with AbD14533.1 and corresponding secondary antibodies.

3T3 cells and CGNs were fixed with 4% PFA for 15 min, washed, and permeabilized with 0.1% Triton X-100. After blocking with 2% goat serum, cells were first incubated with AbD14533.1 and detected using Cy3-conjugated Streptavidin.

For cell surface immunocytochemical detection of S1PR2, 3T3 cells were incubated with 50 µg/ml AbD14533.1 in serum-free medium containing 0.02% sodium azide for 20 min on ice. Cells were washed and fixed with 0.5% PFA. After blocking (4% fetal calf serum, 2% horse serum, 0.1% cold water fish gelatine, 0.1% casein) on ice, cells were first incubated with biotinylated goat anti-human IgG, biotinylated rabbit anti-goat, and, finally, with Cy3-conjugated Streptavidin.

### Electrophysiology

#### Hippocampus

Acute hippocampal slices were prepared from 40–60 day old (P40–P60) WT C57BL/6 mice or Nogo-A^−/−^ mice according to standard procedures. In brief, mice were anesthetized and decapitated; the brain was quickly transferred into ice-cold carbogenated (95% O_2_, 5% CO_2_) artificial cerebrospinal fluid (ACSF). Hippocampi were cut with a vibratome (400 µm; VT 1000S; Leica). The ACSF used for electrophysiological recordings contained 125 mM NaCl, 2 mM KCl, 1.25 mM NaH_2_PO_4_, 1 mM MgCl_2_, 26 mM NaHCO_3_, 2 mM CaCl_2_, 25 mM glucose. Recordings were done at 32°C.

Blockade of S1PR2 was achieved by incubation of acute slices with JTE-013, blockade of S1PR1, 3, 4, 5 with FTY-720 and blockade of S1PR1, 3 with VPC-23019, respectively. The inhibitors were dissolved in DMSO and freshly added at a final concentration of 5 µM, 1 µM, and 0.1 µM, respectively, to the carbogenated ACSF. The DMSO overall concentration in the ACSF was kept at 0.01%. As control DMSO alone was added. In order to compare the data with previous experiments silicon tubing was used, and pre-washed with ACSF containing BSA (0.1 mg/ml). The slices were pre-incubated for 1 h (or 10 min for the experiments in which JTE-013 and 11c7 were combined) with the inhibitor or DMSO as control in an incubation chamber maintaining a constant flow of the solution. During the experiments the inhibitor was also around. For the electrophysiological recordings, the perfusion rate in the recording chamber was constantly kept at 1.5 ml/min.

After placing the slices in a submerged recording chamber field, excitatory postsynaptic potentials (fEPSPs) were recorded in the stratum radiatum of the CA1 region with a glass micropipette (resistance 3–15 MΩ filled with 3 M NaCl at a depth of ∼100 µm. Monopolar tungsten electrodes were used for stimulating the Schaffer collaterals at a frequency of 0.1 Hz. Stimulation was set to elicit a fEPSP with a slope of ∼40%–50% of maximum for LTP recordings. After 20 min baseline stimulation LTP was induced by applying theta-burst stimulation (TBS), in which a burst consisted of four pulses at 100 Hz. These were repeated 10 times in 200 ms intervals (5 Hz). Three such trains were used to induce LTP at 0.1 Hz. Basic synaptic transmission and presynaptic properties were analyzed *via* I/O measurements and paired pulse facilitation. The I/O measurements were performed either by application of a defined value of current (25–250 µA in steps of 25 µA) or by adjusting the stimulus intensity to a certain current eliciting a fiber volley (FV) of desired voltage. Paired pulse facilitation was performed by applying a pair of two stimuli in different inter-stimulus-intervals (ISIs), ranging from 10, 20, 40, and 80 to 160 ms. Data were collected, stored, and analyzed with LABVIEW software (National Instruments). The initial slope of fEPSPs elicited by stimulation of the Schaffer collaterals was measured over time, normalized to baseline, and plotted as average ± SEM.

#### Motor cortex

For LTP measurements in the motor cortex [11], coronal slices containing the forelimb are of M1 (1–2 mm anterior to the bregma [63]), and were prepared from adult Sprague Dawley rats (180–220 g). JTE-013 concentrations were used according to protocols used for hippocampal slices and added to the ACSF: 126 mM NaCl, 3 mM KCl, 1.25 mM NaH_2_PO_4_, 26 mM NaHCO_3_, 1 mM MgSO_4_, 2 mM CaCl_2_, and 10 mM glucose, bubbled with a 95% O_2_, 5% CO_2_ mixture at 33±0.5°C). To allow optimal JTE-013 penetration, responses were recorded from the slice superface of layer II/III within M1. Basic synaptic transmission was analyzed with I/O analysis. I/O measurements were conducted by applying a value of current, which elicited a minimal (threshold) evoked response (0.2–0.3 mV). I/O curves were obtained by averaging field potential peak amplitudes of three responses to stimuli of two, three, four, and five times the threshold response. To elicit the maximum amplitude that could be evoked, we used a stimulation intensity of 25× threshold [64],[65]. For baseline measurements, stimulus intensity was adjusted to produce responses 40%–50% of the maximum amplitude. For data analysis, we computed the amplitude of the field potential response because it serves as a measure of the population excitatory synaptic response [65], reflects a monosynaptic current sink [66], and correlates well with the intracellular excitatory postsynaptic response evoked in this pathway [67]. Measurement of the field potential slope, as routinely used, e.g., in the hippocampus, has not been used for neocortical field potential responses due to the interference of the response's initial part by variable nonsynaptic components [68]. After 20 min of baseline stimulation, focal and transient reduction of y-aminobuturic acid-A (GABA) inhibition at the recording site was produced by applying bicuculline methiodide (3.3 mM, Tocris Bioscience) from a micropipette by touching the tip to the slice surface within 100 µm of the recording microelectrode for 15–60 s. The pipette was removed when the amplitude of test responses increased 50%–100% of baseline [66]. Immediately after bicuculline application, LTP was attempted by delivering TBS at double baseline stimulation intensity. LTP induction was attempted by using TBS, which consisted of 10 trains of stimuli at 5 Hz. Each train was composed of four pulses at 100 Hz. This sequence was delivered every 10 s for a total of five presentations. LTP was recorded for at least 20 min after it reached a stable plateau. TBS was induced until responses were saturated. Pathways were considered saturated if the difference between two subsequent states of LTP were not significantly different [68]. Maximum LTP was calculated as a percentage of baseline, plotted as average ± SEM and analyzed by a Student's t-test. Data were collected, stored, and analyzed with LABVIEW (National Instruments) and MATLAB (MathWorks) software.

## Supporting Information

Figure S1 **S1PR2 expression in 3T3 fibroblasts and immature cerebellar granule cells.** (A,B) Immunofluorescence staining of 3T3 cells (A) and P8 cerebellar granule cell with neurite and growth cone (B) for S1PR2, nuclei (DAPI), and F-Actin (Phalloidin-Alexa488). Scale bars: 50 µm.
(TIF)

Figure S2 **Purity of plasma membrane preparations and specificity of custom-made S1PR2 antibody Ab14533.1.** (A) Western Blot analysis of 3T3 plasma membrane preparations reveals non-detectable amount of EEA1-positive endosomal membranes, but high content of Pan-CDH-positive plasma membrane fractions compared to whole cell lysates. MP, membrane preparations; L, whole cell lysate. (B) Ab14533.1 detects S1PR2 in whole brain tissue extracts. Protein expression is higher in embryonic stages (E11.5, E14.5) than in adult animals. S1PR2 signals are strongly decreased when challenged in a competition assay with the immunogenic peptide (P). (C) Immunohistochemical analysis of S1PR2 in the adult motor cortex (compare to Figure 1E and 1F) shows abolished S1PR2 detection using the same peptide competition assay.
(TIF)

Figure S3 **Blockade of S1PR1, 3, 4, and/or 5 has no effect on Nogo-A-Δ20-mediated cell spreading inhibition.** (A) 3T3 fibroblasts were plated on different concentrations of a Nogo-A-Δ20 substrate in the presence of increasing concentrations of JTE-013 versus vehicle (DMSO). (B) 3T3 fibroblasts were plated on a Nogo-A-Δ20 substrate in the presence of the following pharmacological inhibitors: W146 for S1PR1, VPC-23019 for S1PR1 and 3, and FTY-720 for S1PR1, 3, 4, and 5. DMSO was used as control. A function-blocking anti-S1PR5 antibody had no effect on Nogo-A-Δ20-induced inhibition when compared to anti-BrdU control. (C) 3T3 fibroblasts were plated on a Nogo-A-Δ20 substrate in the presence of JTE-013 in different combinations with VPC-23019, W146 and/or anti-S1PR5. DMSO was used as control. Data shown are means ± SEM (*n* = 3–4 experiments; *p<0.05, **p<0.01, ***p<0.001).
(TIF)

Figure S4 **Knockdown efficacy of S1PR2, G_q_, G_12_, G_13_, and LARG.** (A) Quantitative RT-PCR analysis of S1PR2 expression in 3T3 cells stably expressing *S1pr2* shRNA (sh-*S1pr2*) versus control vector (sh-Vec) revealed an ∼93% knockdown. (B) FACS analysis of S1PR2 expression in 3T3 cells stably expressing sh-*S1pr2* or sh-Vec using the Ab14533.1 antibody. (C) Quantitative RT-PCR analysis of 3T3 cells treated with siRNA targeting *G_q_*, *G_12_*, *G_13_*, or *Larg* for 72 h. Scrambled siRNA (ctrl) was used as control. Relative quantification of knockdown efficacy: *G_12_* (∼77%), *G_13_* (∼78%), *G_q_* (∼79%), and *Larg* (83%). (D) FACS analysis of G_13_ expression in *G_13_* versus ctrl siRNA-treated 3T3 cells. (E) Quantitative RT-PCR analysis of E19 rat cortical neurons treated at DIV4 with siRNA targeting *G_12_* or *G_13_* for 72 h. Scrambled siRNA (ctrl) was used as control. Relative quantification of knock-down efficacy: *G_12_* (39%), *G_13_* (42%). (F) FACS analysis of G_13_ expression in *G_13_* versus ctrl siRNA-treated E19 cortical neurons. (G) FACS analysis of LARG expression in *Larg* versus ctrl siRNA-treated 3T3 cells. Histograms from one representative experiment are shown. Data shown are means ± SEM (*n* = 3 experiments).
(TIF)

Figure S5 **S1PR2 blockade has no effect on Nogo-66- and Aggrecan-mediated inhibition of neurite outgrowth.** (A,B) Mean neurite length quantification of P5–8 CGNs treated with JTE-013 or DMSO and plated on a Nogo-66 (A) or Aggrecan (B) versus ctrl (PLL) substrate. Data shown are means ± SEM (*n* = 4 replicates).
(TIF)

Figure S6 **Pharmacological inhibition of S1PR1 and 3 or S1PR1, 3, 4, and 5 does not increase hippocampal LTP.** (A,B) WT hippocampal slices were treated with VPC-23019 (*n* = 7) (A) or FTY-720 (*n* = 8) (B) to block S1PR1 and 3 or S1PR1, 3, 4 and 5, respectively. DMSO was used as control in (A) (*n* = 11) and (B) (*n* = 9). No significant differences in LTP could be observed between VPC-23019, FTY-720 and DMSO treatment. (C) PPF revealed no alterations in VPC-23019- (*n* = 5) or FTY-720- (*n* = 7) versus DMSO- (*n* = 7) treated slices. (D) No significant difference in LTP could be observed in S1PR2^−/−^ (*n* = 11) versus WT (*n* = 12) mice. (E) Input-output strength revealed no alterations in S1PR2^−/−^ (*n* = 8) versus WT (*n* = 12) mice. (F) PPF revealed no alterations in S1PR2^−/−^ (*n* = 11) versus WT (*n* = 13) mice. (G) No significant difference in hippocampal long-term depression (LTD) could be observed between JTE-013- (*n* = 4) versus DMSO- (*n* = 5) treated WT slices. Arrows indicate the onset of theta-burst (A,B,D) or low frequency (G) stimulation. Data shown are means ± SEM. *n* indicates the number of mice used.
(TIF)

## Abbreviations

aa: amino acid
CNS: central nervous system
ECL: extracellular loop
GPCR: G protein-coupled receptor
I/O: input-output
LTP: long-term potentiation
MEF: mouse embryonic fibroblast
MW: molecular weight
NgR1: Nogo-66 receptor 1
Nogo-A-ext: Δ20-containing extracellular N-terminal domain of Nogo-A
PirB: paired immunoglobulin-like receptor B
PPF: paired-pulse facilitation
S1P: sphingosine 1-phosphate
S1PR2: sphingosine 1-phosphate receptor 2
SEM: standard error of the mean
siRNA: small interfering RNA
SphK: sphingosine kinase
WT: wild type
