# Correction: The Sphingolipid Receptor S1PR2 Is a Receptor for Nogo-A Repressing Synaptic Plasticity

**DOI:** 10.1371/journal.pbio.1001818

**Published:** 2014-02-28

**Authors:** The PLOS Biology Staff

In the legend for Figure 4, there is a misspelling. The correct spelling is: 50 µm. The authors have provided a corrected version here.

**Figure 4:**
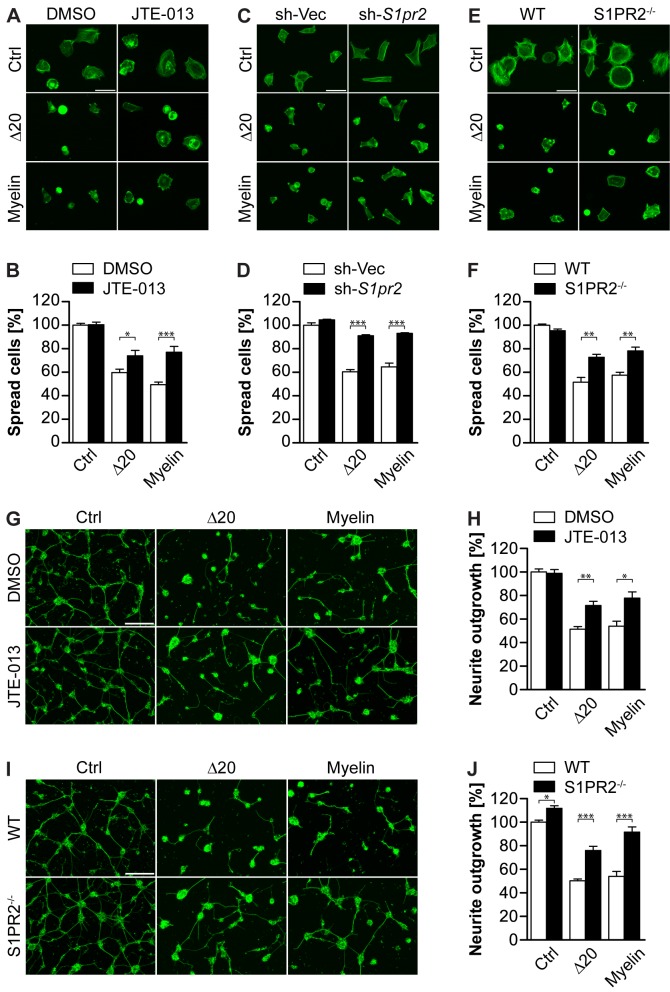
S1PR2 mediates Nogo-A-Δ20- and myelin-induced inhibition of cell spreading and neurite outgrowth. (A,C) Representative pictures of 3T3 fibroblasts treated with JTE-013 or vehicle (DMSO) (A), or stably carrying a S1pr2 shRNA (sh-S1pr2) or empty vector (sh-Vec) construct (C) and plated on control, Nogo-A-Δ20 or myelin substrates. (B,D) Cell spreading quantification of (A) and (C). (E) Representative pictures of MEFs isolated from WT or S1PR2^−/−^ mice and plated on control, Nogo-A- Δ20, or myelin substrates. (F) Cell spreading quantification of (E). Cells were stained with Alexa488-conjugated Phalloidin in (A, C, and E). (G,I) Representative pictures of P5–8 cerebellar granule neurons treated with JTE-013 or DMSO (G), or isolated from S1PR2^−/−^ or WT mice (I) and plated on PLL (ctrl), Nogo-A-Δ20 or myelin substrates. (H,J) Normalized mean neurite length per cell quantification of (G) and (I). Neurons were stained with βIII-Tubulin in (G) and (I). Data shown are means ± SEM (n  =  3–6 experiments; *p<0.05, **p<0.01, ***p<0.001). Scale bars: 50 µm.

Figure 5 is incorrect. Figure panel 5H was an inadvertent duplication of the figure panel 5G. Figures 5G and H show ELISA quantifications of extra- (EC) and intracellular (IC) S1P levels in 3T3 cells (G) and cerebellar granule neurons (H) before and after 30 and 60 min incubation with Nogo-A-Δ20. The authors have provided a corrected version here.

**Figure 5:**
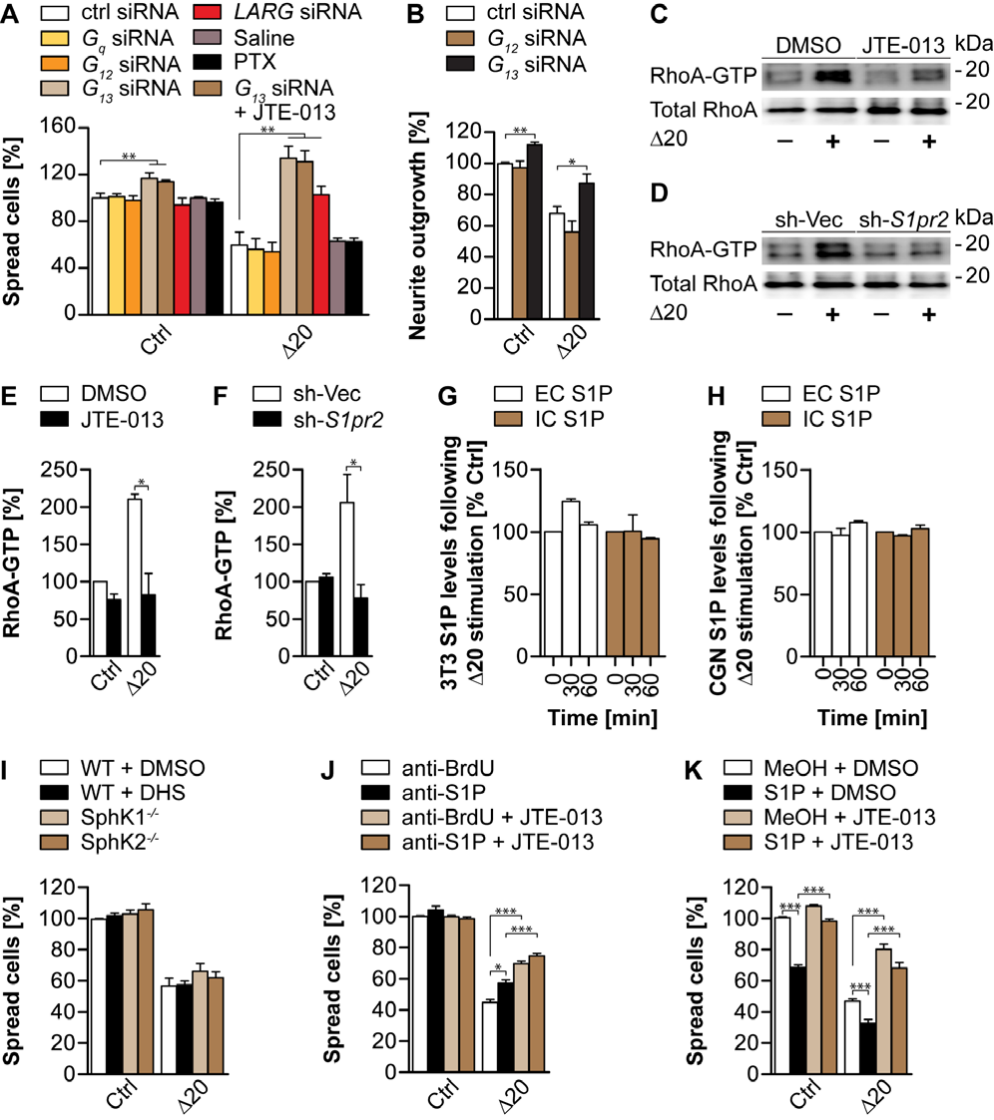
Nogo-A-Δ20 inhibition is mediated via the G13-LARG-RhoA signaling axis and can be modulated by exogenous S1P. (A) 3T3 cells transfected with siRNAs against G12, G13, Gq, or Larg, or control (ctrl) siRNA were replated on a Nogo-A-Δ20 substrate and assessed for cell spreading. Gi/o was blocked with Pertussis Toxin (PTX) for which saline was used as control. JTE-013 was co-applied to G13-siRNA-treated cells to investigate a cumulative effect. (B) Transfection of DIV4 E19 cortical neurons with siRNA against G13 but not G12 similarly rescued Nogo-A-Δ20-induced neurite outgrowth inhibition. (C,D) Nogo-A-Δ20-induced RhoA activation was assessed in JTE-013- versus DMSO-treated cells (C) or in cells carrying a stable knockdown of S1PR2 (sh-S1pr2) versus control vector (sh-Vec) (D). (E,F) Relative quantification of (C) and (D), respectively. (G,H) Competitive ELISA quantifications of extra- (EC) and intracellular (IC) S1P levels in 3T3 cells (G) and cerebellar granule neurons (H) before and after 30 and 60 min incubation with Nogo-A-Δ20. (I) Quantification of Nogo-A-Δ20-mediated cell spreading inhibition in the presence of the SphK-specific blocker D,L-threo-dihydrosphingosine (DHS) or in SphK1−/− or SphK2−/− MEFs. (J,K) 3T3 cells were plated on a Nogo-A-Δ20 substrate in the presence of the function blocking anti-S1P antibody Sphingomab (J) or of exogenous S1P (K) and assessed for cell spreading. Co-application of JTE-013 significantly reversed the modulatory effects obtained by S1P (K) but not anti-S1P (J). Anti-BrdU antibody or methanol was used as control in (J) and (K). Data shown are means ± SEM (n  =  3–6 experiments; *p<0.05, **p<0.01, ***p<0.001).
