# Supplementary figures and images for: The Sphingolipid Receptor S1PR2 Is a Receptor for Nogo-A Repressing Synaptic Plasticity
Source: PLoS Biol. 2014 Jan 14;12(1):e1001763. doi: 10.1371/journal.pbio.1001763 (PMC3891622; doi:10.1371/journal.pbio.1001763)

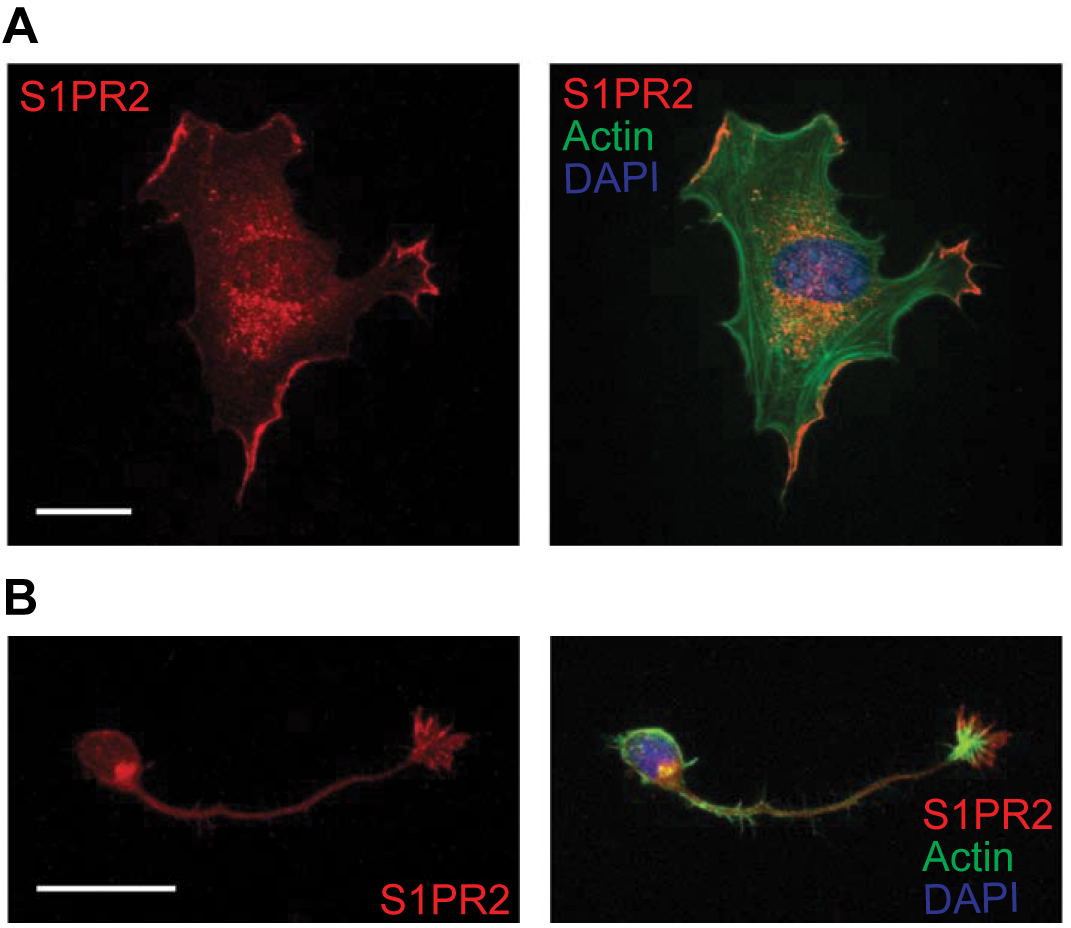

Supplement: Figure S1 — S1PR2 expression in 3T3 fibroblasts and immature cerebellar granule cells. (A,B) Immunofluorescence staining of 3T3 cells (A) and P8 cerebellar granule cell with neurite and growth cone (B) for S1PR2, nuclei (DAPI), and F-Actin (Phalloidin-Alexa488). Scale bars: 50 µm. (TIF) [file pbio.1001763.s001.tif]

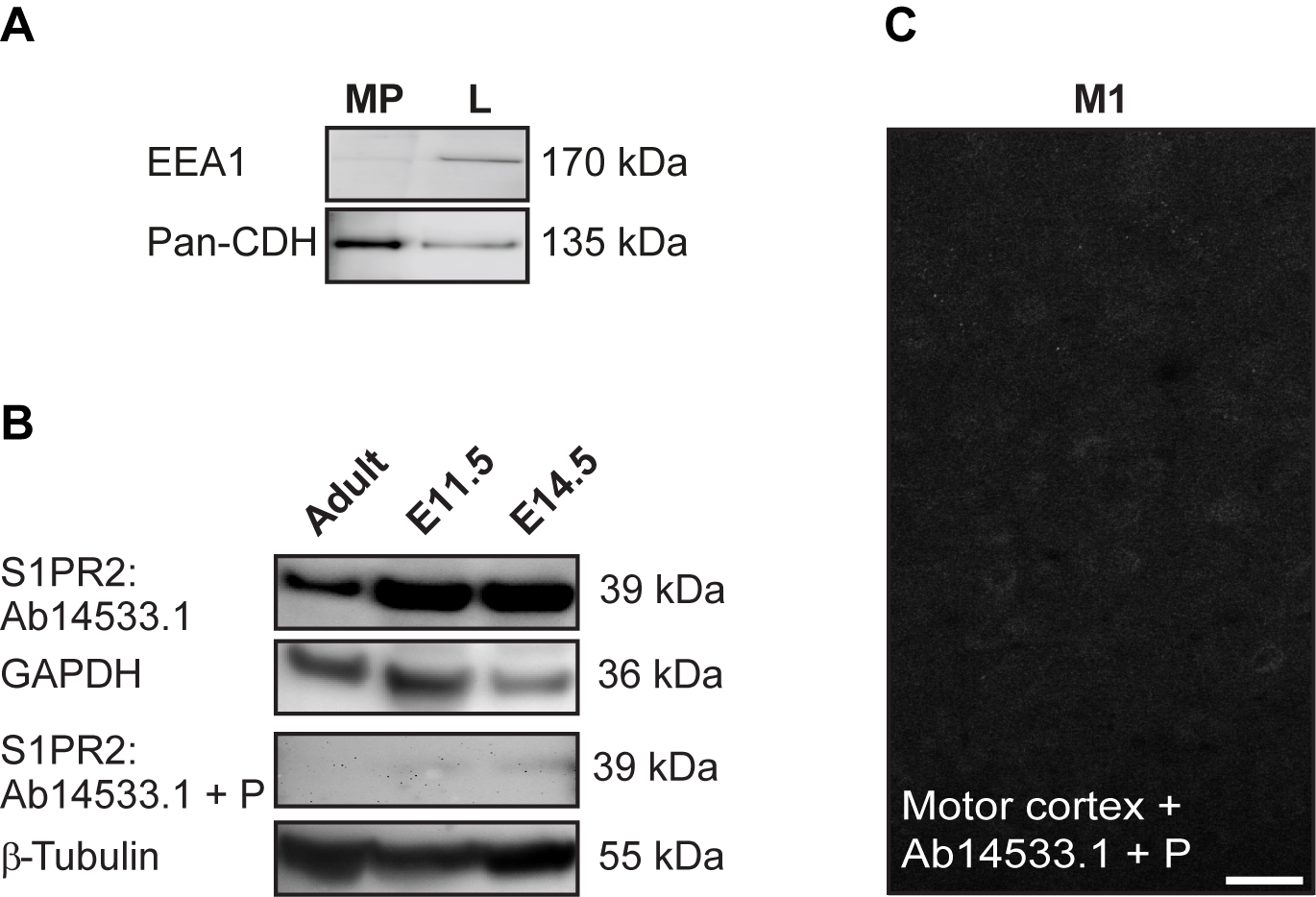

Supplement: Figure S2 — Purity of plasma membrane preparations and specificity of custom-made S1PR2 antibody Ab14533.1. (A) Western Blot analysis of 3T3 plasma membrane preparations reveals non-detectable amount of EEA1-positive endosomal membranes, but high content of Pan-CDH-positive plasma membrane fractions compared to whole cell lysates. MP, membrane preparations; L, whole cell lysate. (B) Ab14533.1 detects S1PR2 in whole brain tissue extracts. Protein expression is higher in embryonic stages (E11.5, E14.5) than in adult animals. S1PR2 signals are strongly decreased when challenged in a competition assay with the immunogenic peptide (P). (C) Immunohistochemical analysis of S1PR2 in the adult motor cortex (compare to Figure 1E and 1F) shows abolished S1PR2 detection using the same peptide competition assay. (TIF) [file pbio.1001763.s002.tif]

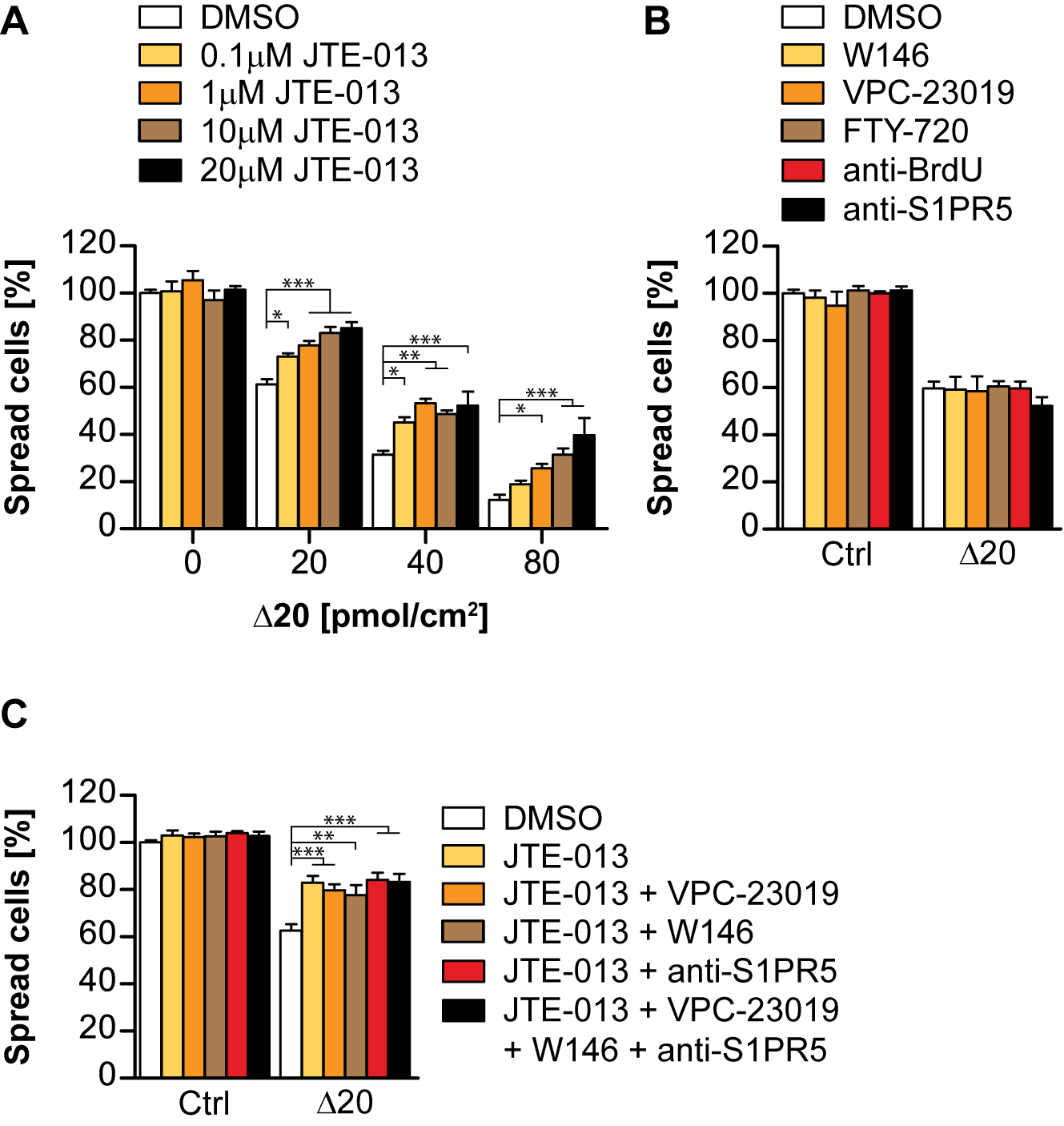

Supplement: Figure S3 — Blockade of S1PR1, 3, 4, and/or 5 has no effect on Nogo-A-Δ20-mediated cell spreading inhibition. (A) 3T3 fibroblasts were plated on different concentrations of a Nogo-A-Δ20 substrate in the presence of increasing concentrations of JTE-013 versus vehicle (DMSO). (B) 3T3 fibroblasts were plated on a Nogo-A-Δ20 substrate in the presence of the following pharmacological inhibitors: W146 for S1PR1, VPC-23019 for S1PR1 and 3, and FTY-720 for S1PR1, 3, 4, and 5. DMSO was used as control. A function-blocking anti-S1PR5 antibody had no effect on Nogo-A-Δ20-induced inhibition when compared to anti-BrdU control. (C) 3T3 fibroblasts were plated on a Nogo-A-Δ20 substrate in the presence of JTE-013 in different combinations with VPC-23019, W146 and/or anti-S1PR5. DMSO was used as control. Data shown are means ± SEM (n = 3–4 experiments; *p<0.05, **p<0.01, ***p<0.001). (TIF) [file pbio.1001763.s003.tif]

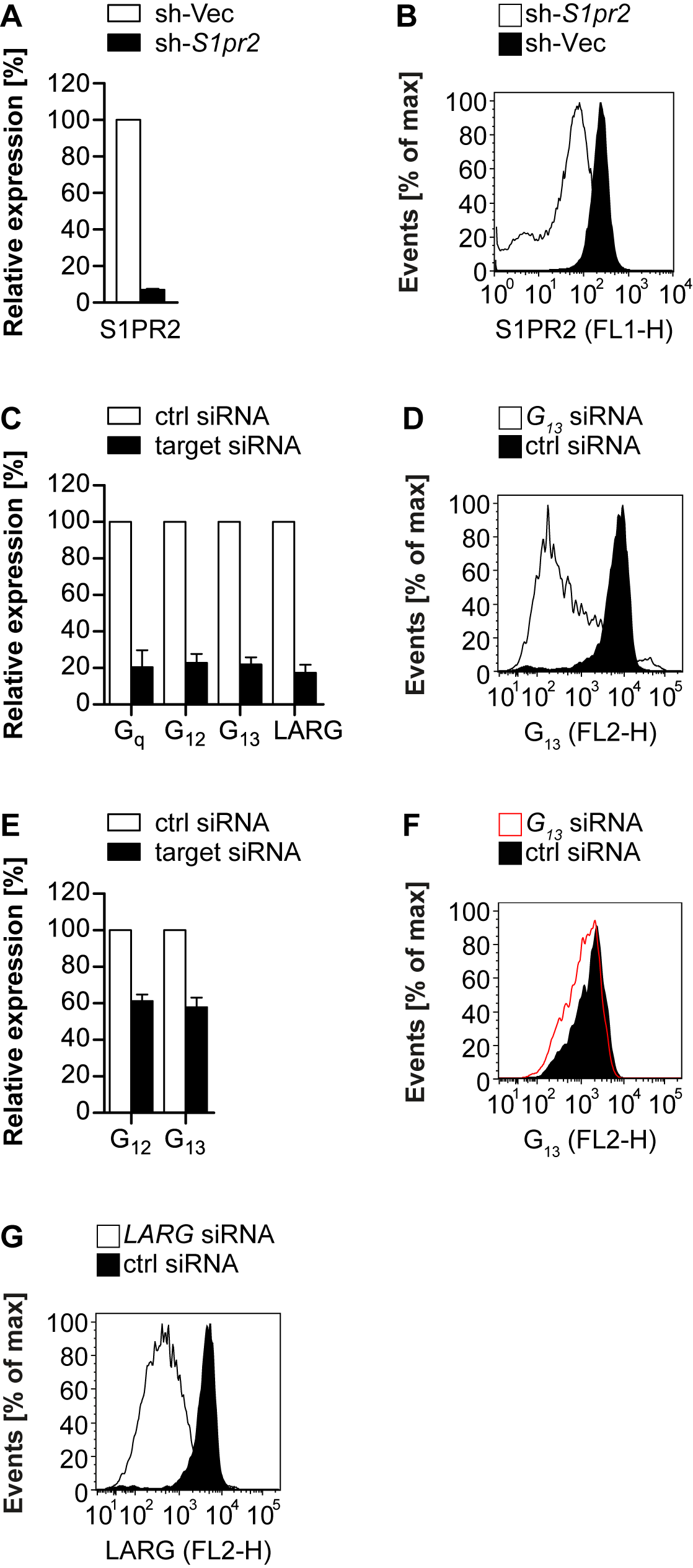

Supplement: Figure S4 — Knockdown efficacy of S1PR2, Gq, G12, G13, and LARG. (A) Quantitative RT-PCR analysis of S1PR2 expression in 3T3 cells stably expressing S1pr2 shRNA (sh-S1pr2) versus control vector (sh-Vec) revealed an ∼93% knockdown. (B) FACS analysis of S1PR2 expression in 3T3 cells stably expressing sh-S1pr2 or sh-Vec using the Ab14533.1 antibody. (C) Quantitative RT-PCR analysis of 3T3 cells treated with siRNA targeting Gq, G12, G13, or Larg for 72 h. Scrambled siRNA (ctrl) was used as control. Relative quantification of knockdown efficacy: G12 (∼77%), G13 (∼78%), Gq (∼79%), and Larg (83%). (D) FACS analysis of G13 expression in G13 versus ctrl siRNA-treated 3T3 cells. (E) Quantitative RT-PCR analysis of E19 rat cortical neurons treated at DIV4 with siRNA targeting G12 or G13 for 72 h. Scrambled siRNA (ctrl) was used as control. Relative quantification of knock-down efficacy: G12 (39%), G13 (42%). (F) FACS analysis of G13 expression in G13 versus ctrl siRNA-treated E19 cortical neurons. (G) FACS analysis of LARG expression in Larg versus ctrl siRNA-treated 3T3 cells. Histograms from one representative experiment are shown. Data shown are means ± SEM (n = 3 experiments). (TIF) [file pbio.1001763.s004.tif]

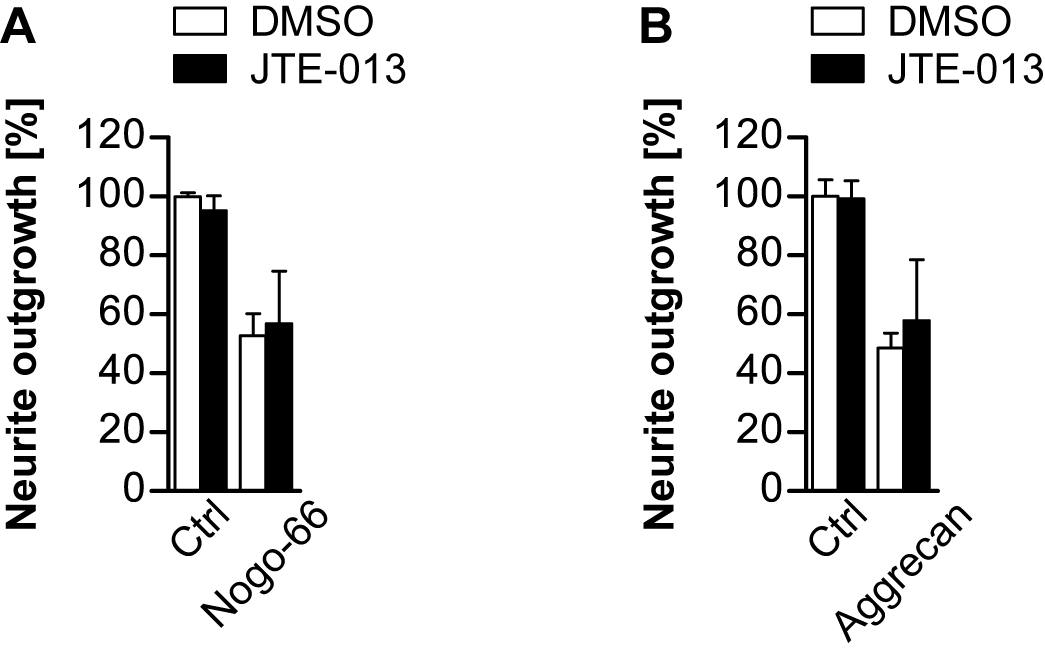

Supplement: Figure S5 — S1PR2 blockade has no effect on Nogo-66- and Aggrecan-mediated inhibition of neurite outgrowth. (A,B) Mean neurite length quantification of P5–8 CGNs treated with JTE-013 or DMSO and plated on a Nogo-66 (A) or Aggrecan (B) versus ctrl (PLL) substrate. Data shown are means ± SEM (n = 4 replicates). (TIF) [file pbio.1001763.s005.tif]

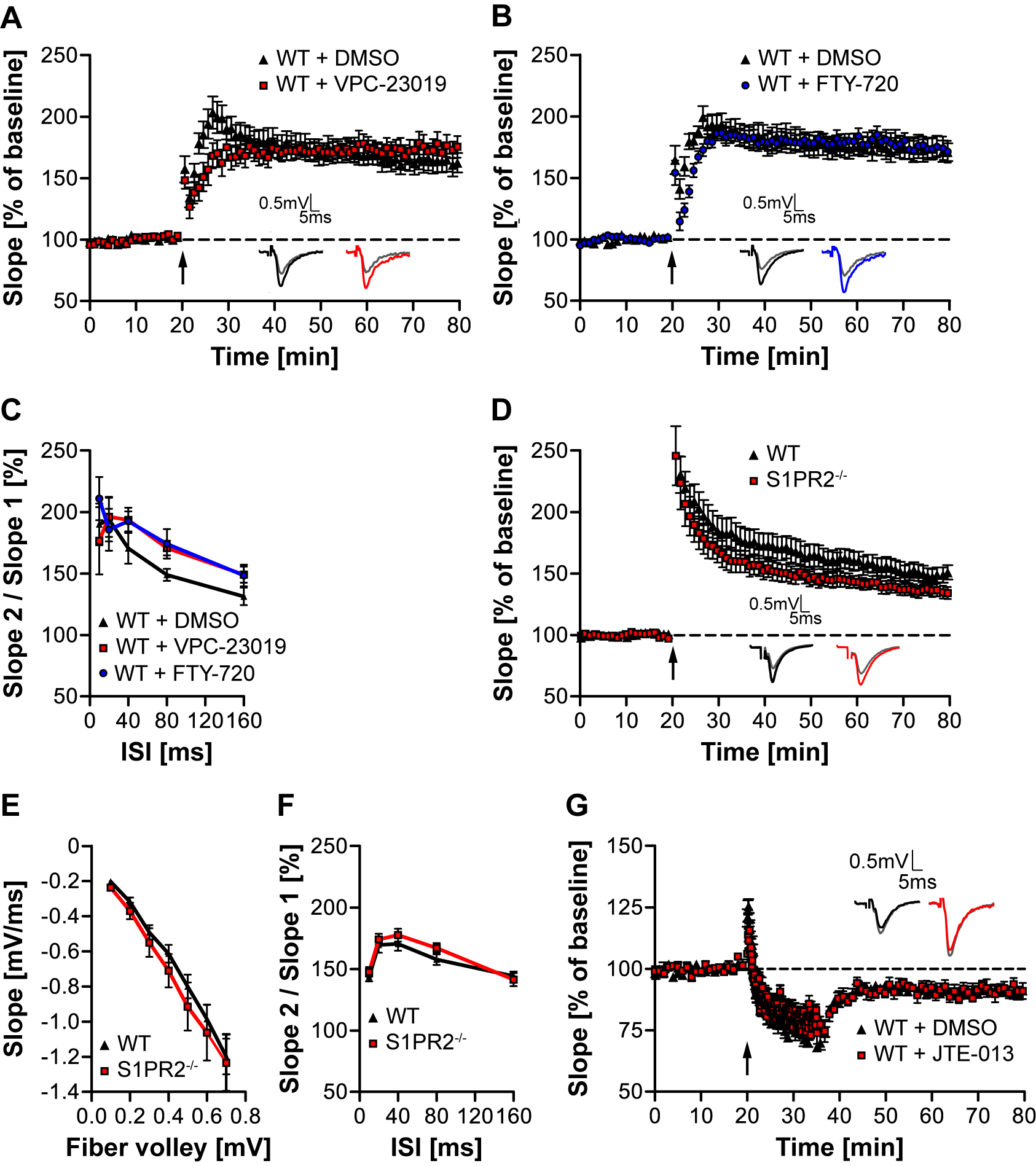

Supplement: Figure S6 — Pharmacological inhibition of S1PR1 and 3 or S1PR1, 3, 4, and 5 does not increase hippocampal LTP. (A,B) WT hippocampal slices were treated with VPC-23019 (n = 7) (A) or FTY-720 (n = 8) (B) to block S1PR1 and 3 or S1PR1, 3, 4 and 5, respectively. DMSO was used as control in (A) (n = 11) and (B) (n = 9). No significant differences in LTP could be observed between VPC-23019, FTY-720 and DMSO treatment. (C) PPF revealed no alterations in VPC-23019- (n = 5) or FTY-720- (n = 7) versus DMSO- (n = 7) treated slices. (D) No significant difference in LTP could be observed in S1PR2−/− (n = 11) versus WT (n = 12) mice. (E) Input-output strength revealed no alterations in S1PR2−/− (n = 8) versus WT (n = 12) mice. (F) PPF revealed no alterations in S1PR2−/− (n = 11) versus WT (n = 13) mice. (G) No significant difference in hippocampal long-term depression (LTD) could be observed between JTE-013- (n = 4) versus DMSO- (n = 5) treated WT slices. Arrows indicate the onset of theta-burst (A,B,D) or low frequency (G) stimulation. Data shown are means ± SEM. n indicates the number of mice used. (TIF) [file pbio.1001763.s006.tif]
